# Microbiome and metabolome analyses reveal significant alterations of gut microbiota and bile acid metabolism in ETEC-challenged weaned piglets by dietary berberine supplementation

**DOI:** 10.3389/fmicb.2024.1428287

**Published:** 2024-06-25

**Authors:** Xiaoyan Nie, Qi Lu, Yucheng Yin, Zhentao He, Yinshan Bai, Cui Zhu

**Affiliations:** ^1^School of Life Science and Engineering, Foshan University, Foshan, China; ^2^Guangdong Province Doctoral Workstation, Shanwei Xinsheng Leisure Agriculture Co., Ltd, Shanwei, China

**Keywords:** weaned piglet, enterotoxigenic *Escherichia coli*, tight junction proteins, berberine, gut microbiota, bile acids

## Abstract

This study mainly investigated the effects of berberine (BBR) on the bile acid metabolism in gut-liver axis and the microbial community in large intestine of weaned piglets challenged with enterotoxigenic *Escherichia coli* (ETEC) by microbiome and metabolome analyses. Sixty-four piglets were randomly assigned to four groups including Control group, BBR group, ETEC group, and BBR + ETEC group. Dietary BBR supplementation upregulated the colonic mRNA expression of *Occludin*, *Claudin-5*, trefoil factor 3 (*TFF3*), and interleukin (*IL*)*-10*, and downregulated colonic *IL-1β* and *IL-8* mRNA expression in piglets challenged with ETEC K88 (*p* < 0.05). The hepatic non-targeted metabolome results showed that dietary BBR supplementation enriched the metabolic pathways of primary bile acid biosynthesis, tricarboxylic acid cycle, and taurine metabolism. The hepatic targeted metabolome analyses showed that BBR treatment increased the hepatic concentrations of taurocholic acid (TCA) and taurochenodeoxycholic acid (TDCA), but decreased the hepatic cholic acid (CA) concentration (*p* < 0.05). Further intestinal targeted metabolome analyses indicated that the deoxycholic acid (DCA), hyocholic acid (HCA), 7-ketodeoxycholic acid (7-KDCA), and the unconjugated bile acid concentrations in ileal mucosa was decreased by dietary BBR treatment (*p* < 0.05). Additionally, BBR treatment significantly upregulated the hepatic holesterol 7 α-hydroxylase (*CYP7A1*) and sterol 27-hydroxylase (*CYP27A1*) mRNA expression, and upregulated the ileal mRNA expression of farnesoid X receptor (*FXR*) and apical sodium-dependent bile acid transporter (*ASBT*) as well as the colonic mRNA expression of *FXR*, fibroblast growth factor19 (*FGF19*), takeda G protein-coupled receptor 5 (*TGR5*) and organic solute transporters beta (*OST-β*) in piglets (*p* < 0.05). Moreover, the microbiome analysis showed that BBR significantly altered the composition and diversity of colonic and cecal microbiota community, with the abundances of Firmicutes (phylum), and *Lactobacillus* and *Megasphaera* (genus) significantly increased in the large intestine of piglets (*p* < 0.05). Spearman correlation analysis showed that the relative abundances of *Megasphaera* (genus) were positively correlated with *Claudin-5*, *Occludin*, *TFF3*, and hepatic TCDCA concentration, but negatively correlated with hepatic CA and glycocholic acid (GCA) concentration (*p* < 0.05). Moreover, the relative abundances of Firmicute (phylum) and *Lactobacillus* (genus) were positively correlated with hepatic TCDCA concentration (*p* < 0.05). Collectively, dietary BBR supplementation could regulate the gut microbiota and bile acid metabolism through modulation of gut-liver axis, and attenuate the decreased intestinal tight junction expression caused by ETEC, which might help maintain intestinal homeostasis in weaned piglets.

## 1 Introduction

Enterotoxigenic *Escherichia coli* (ETEC) represents the predominant cause for post-weaning diarrhea in piglets (Dubreuil et al., 2016), which would lead to impaired intestinal health, decreased growth performance and increased mortality in piglets. Previous study has shown that ETEC infection caused the dysbiosis of gut microbiota and alterations of bile acid metabolism, which could mediate the ETEC-induced diarrhea in piglets (Bin et al., 2018). Bile acids are important signaling metabolites that participate in the regulation of metabolism and inflammation through dynamic interactions with the host and microbiota (Chen et al., 2019; Pi et al., 2023). In addition, increasing evidence has shown the crosstalk between gut microbiota and bile acids are essential for maintaining intestinal barrier function and intestinal health of the host (Nie et al., 2015; Lin et al., 2019; Zhai et al., 2021). Thus, the modulation of microbiota-bile acid axis may represent an important target for potential nutritional strategies to restore the intestinal homeostasis in piglets with ETEC infection.

Berberine (BBR) is an isoquinoline alkaloid isolated from various herb plants with strong anti-inflammatory activities and displays broad antibacterial spectrum against many pathogens (Imenshahidi and Hosseinzadeh, 2016). Thus, BBR has been widely used in the treatment of diarrhea and intestinal inflammation in human for a long period (Song et al., 2020). Previous studies in piglets have demonstrated that dietary BBR supplementation could enhance intestinal epithelial barrier (Zhu et al., 2023), and effectively alleviate the intestinal oxidative injury (Tang et al., 2021). Notably, the beneficial effects of BBR on gut health may be related to the alterations of gut microbiota composition and its metabolite bile acid concentration (Guo et al., 2016). Bile acids have been recognized to play important roles in regulating the barrier integrity and alleviating the intestinal inflammation through the interactions with gut microbiota and host responses by activating its receptors including farnesoid × receptor (FXR) and takeda G proteincoupled receptor 5 (TGR5) (Lin et al., 2019; Zhai et al., 2021). However, it remained unclear whether BBR could restore the dysbiosis of gut microbiota and abnormal bile acid profiles induced by ETEC for maintaining intestinal homeostasis through modulation of the gut microbiota-bile acid axis in piglets.

Metabolome is an emerging technology that reveals the dynamic changes and regulation of the overall metabolites of an organism by studying small molecules (Bracewell-Milnes et al., 2017). Microbiome mainly explores the crosstalk and interaction between gut microbiota and their environment with the host (Vernocchi et al., 2016). Combined analyses of metabolome and microbiome are recognized as a powerful tool to provide insights into how gut microbiota affect host metabolic status and phenotypic changes through microbial metabolites (Liu et al., 2017). Therefore, this study was carried out to investigate the effects of dietary BBR supplementation on the changes of bile acid metabolism and gut microbial community in weaning piglets after ETEC challenge via both non-targeted and targeted metabolome and microbiome analyses, and to determine the expression of intestinal tight junctions and cytokines, as well the genes involved in the bile acid metabolism of gut-liver axis.

## 2 Materials and methods

### 2.1 Animal, diets and experimental design

The experimental protocols and procedures performed in this study were approved by the Animal Care and Use Committee of Foshan University (FOSU2022005). A total of 64 21-day-old crossbred weaned barrows (initial body weight of 6.75 ± 0.19 kg, Duroc × Yorkshire × Landrace) were selected in a 2 × 2 factorial arrangement and randomly assigned to the following four treatment groups including the Control group, BBR group, ETEC group, and BBR + ETEC group. The piglets were fed with either the basal diets (Supplementary Table S1) or the basal diets supplemented with 250 mg/kg BBR from a commercial company (Shanxi Sciphar Natural Products Co., Ltd., Xi’an, Shanxi, China). The basal diets were formulated to meet or exceed nutrient requirements for piglets at 7–11 kg recommended by National Research Council (NRC, 2012). On days 15 and 17, the piglets in ETEC group and BBR + ETEC groups were orally challenged with either 10 mL ETEC K88 solution (serotype O140: K91: K88ac, 1 × 10^9^ CFU/mL), while those in the other two groups were treated with the same amount of sterile PBS as previously described (Zhu et al., 2023). All piglets had free access to feed in mash form and water during the whole experimental period.

### 2.2 Sample collection

On the morning of d 18 of the experiment, one piglet with similar average body weight was randomly selected from each replicate for sample collection. The piglets were euthanized with sodium pentobarbital (50 mg/kg body weight) via anterior vein, and harvested for intestine and liver samples. The digesta of colon and cecum were collected for microbial analysis. After flushing with ice-cold saline, the ileal and colonic mucosa samples were scraped gently by a sterile glass slide as previously described (Zhu et al., 2023). All the samples were immediately snap-frozen in liquid nitrogen followed by storage at −80°C until further analyses.

### 2.3 RNA extraction, cDNA synthesis and quantitative real time (RT)-PCR

Total RNA was isolated from the liver, ileal and colonic mucosa samples using TRIzol reagent (Invitrogen, Carlsbad, CA, USA) according to the manufacturer’s instructions. The purity and concentration of RNA were determined using a DS-11 spectrophotometer (DeNovix, Wilmington, DE, USA). All samples were checked with A260/280 of 1.8–2.0 and then verified by 1% agarose gel electrophoresis for RNA integrity. Total RNA (1 μg) was used to synthesize cDNA using a reverse transcription kit (Takara, Japan). The cDNA was quantified by real-time fluorescence using iTaq™ Universal SYBR Green Supermix (Bio-Rad, USA) in a QuantStudio 3 Flex real-time system (Applied Biosystems Instruments, Thermo Fisher Scientific, San Jose, CA, USA). The quantitative RT-PCR reactions consisted of 39 cycles of initial denaturation at 95°C for 30 s, followed by denaturation at 95°C for 15 s, annealing at 60°C for 30 s, extension at 72°C for 30 s, and finally extension at 72°C for 5 min. Primers (Table 1) were de-signed using Primer Premier 5.0 software (Applied Biosystems, USA) and then synthesized by Ige Biotech Co. (Guangzhou, China). The internal reference gene β-actin was used as an internal control with three replicates for each sample, and the fold change of the target gene was calculated for each sample using the 2^-∆∆CT^ method (Livak and Schmittgen, 2001).

**Table 1:** Primer sequences for quantitative real-time PCR.

**Genes**^**a**^	**Primer sequences**^**b**^	**Access number**
*CYP7A1*	F-CCGCTTCTGATACCTGTGGA	NM_001005352.3
	R-GGTTTGCTCGGAGGAACTCA	
*CYP8B1*	F-CAAGTTCGACCGCATGTTCC	NM_214426.1
	R-TTATGCCGTGCCTCTCCAAG	
*CYP27A1*	F-GAGGGCAAGTACCCAGTACG	NM_001243304.1
	R-TGACTCTCCTTCCGTGGTGA	
*FXR*	F-TATGAACTCAGGCGAATGCCTGCT	NM_001287412.1
	R-ATCCAGATGCTCTGTCTCCGCAAA	
*TGR5*	F-CCATGCACCCCTGTTGCT	XM_013984487.2
	R-GGTGCTGTTGGGTGTCATCTT	
*SHP*	F-TGCTGCCTGGAGTCCTTATG	XM_003127720.4
	R-ACAGGGCGAAAGAAGAGGTC	
*FGF19*	F-TGAGTACCGTGGCGATCAAG	XM_003122420.3
	R-GCGGATCTCCTCCTCGAAAG	
*OST-β*	F-GGCGTGTGCTAAATGCAGAG	XM_005658570.3
	R-GTTTTCCACACGGCTGTCAC	
*OST-α*	F-TGTACAAGAACACTCGCTGC	NM_001244266.1
	R-GAACACACACACTATCGTGGG	
*ASBT*	F-CCAGAGTGCCTGGATCATCG	NM_001244463.1
	R-GGAGTAACCGGCCAAAGGAA	
*Occludin*	F-GCACCCAGCAACGACAT	XM_005672525
	R-CATAGACAGAATCCGAATCAC	
*ZO-1*	F-AGCCCGAGGCGTGTTT	XM_013993251
	R-GGTGGGAGGATGCTGTTG	
*Claudin-5*	F-CCTTCCTGGACCACAACATC	NM_001161636.1
	R-CACCGAGTCGTACACCTTGC	
*Claudin-1*	F- ACGGCCCAGGCCATCTAC	AJ318102.1
	R- TGCCGGGTCCGGTAGATG	
*TFF3*	F-CAGGATGTTCTGGCTGCTAGTG	NM_001243483.1
	R-GCAGTCCACCCTGTCCTTG	
*IL-1β*	F-CTCCAGCCAGTCTTCATTGTTC	NM_001302388.1
	R-TGCCTGATGCTCTTGTTCCA	
*IL-8*	F-AGGACCAGAGCCAGGAA	NM_213867.1
	R-GTGGAATGCGTATTTATGC	
*IL-10*	F-GGTTGCCAAGCCTTGTCAG	NM_214041
	R-AGGCACTCTTCACCTCCTC	
*β-actin*	F-TCCACCGCAAATGCTTCTAG	AY550069
	R-TGCTGTCACCTTCACCGTTC	

^a^CYP7A71, cholesterol 7α-hydroxylase; CYP8B1, 12a-hydroxylase; CYP27A1, sterol 27-hydroxylase; FXR, farnesoid X receptor; TGR5, takeda G protein-coupled receptor 5; SHP, small heterodimer partner; FGF19, fibroblast growth factor19; OST-β, organic solute transporters beta; OST-*α*, organic solute transporters alpha; ASBT, apical sodium-dependent bile acid transporter; ZO-1, zonula occludens-1; TFF3, trefoil factor 3; IL-1β, interleukin-1β; IL-8, interleukin-8; IL-10, interleukin-10. ^b^F, forward; R, reverse.

### 2.4 Analysis of gut microbial composition and diversity by 16S rRNA sequencing

Total microbial genomic DNA in the colonic and cecal digesta were extracted using a QIAamp fast DNA stool mini kit (Qiagen, Germany). The concentration and purity of extracted DNA were monitored by electrophoresis on 1% agarose gels and spectrophotometry using the NanoDrop 2000 (Thermo Scientific, Wilmington, DE). The V3 to V4 regions of the 16S rRNA gene were amplified with primers 341F (5’-ACTCCTACGGGAGGCAGCAG-3’) and 806R (5’-GGACTACHVGGGTWTCTAAT-3’). The resulting PCR product was purified and quantified using the15 μL of Phusion® High- Fidelity PCR Master Mix (New England Biolabs).Thermal cycling consisted of initial denaturation at 98°C for 1 min, followed by 30 cycles of denaturation at 98°C for 10 s, annealing at 50°C for 30 s, and elongation at 72°C for 30 s and 72°C for 5 min. In consistent with previous study (Zhu et al., 2023), the 16S rRNA sequencing was performed by the Illumina HiSeq 2,500 PE250 platform (Novo-gene Bioinformatics Technology Co., Ltd., Tianjin, China) and bioinformatics analysis protocols of sequencing data was conducted using the QIIME software package (version 1.9.1). The relative abundances of top 10 most abundant bacteria at the phylum and genus levels were calculated using the taxa plugin. The results of α-diversity included Shannon index, Simpson index, Chao1, and Goods_ coverage were provided. Moreover, the β-diversity index, principal coordinate analysis (PCoA) and unweighted pair-group method with arithmetic means (UPGMA) clustering, as well as the analysis of similarities (ANOSIM) were used to evaluate treatment differences in complexity of species diversity. The difference in the relative abundances of microbiota among treatments were further analyzed by linear discriminant analysis effect size (LEfSe) and t-test analysis.

### 2.5 Non-targeted metabolome analysis of liver and data processing

Briefly, 50 mg of frozen liver samples were extracted with 1,000 μL extraction solvent containing methanol, acetonitrile and water (2,2:1, v/v) solution and an internal standard of 20 μL ribitol solution (1 mg/mL). The samples were vortexed for 30 s, and homogenized at 45 Hz for 4 min with an ultrasonic processor, and sonicated for 5 min in ice-water bath. The homogenate and sonicate circle were repeated for 3 times, followed by incubation at −20°C for 1 h and centrifugation at 12,000 × g and 4°C for 15 min. The resulting supernatants were transferred to LC–MS vials and stored at −80°C until the UHPLC-QE Orbitrap/MS analysis. The quality control (QC) sample was prepared by mixing an equal aliquot of the supernatants from all the samples.

The LC–MS/MS analyses were performed using an UHPLC system (1,290, Agilent Technologies, USA) with a UPLC HSS T3 column (2.1 mm × 100 mm, 1.8 μm) coupled to Q Exactive (Orbitrap MS, Thermo Fisher Scientific). The mobile phase A was 0.1% formic acid in water for positive, and 5 mM ammonium acetate in water for negative, and the mobile phase B was acetonitrile. The elution gradient was set as follows: 0 min, 1% B; 1 min, 1% B; 8 min, 99% B; 10 min, 99% B; 10.1 min, 1% B; 12 min, 1% B. The flow rate was 0.5 mL/min. The injection volume was 2 μL. The QE mass spectrometer was used for its ability to acquire MS/MS spectra on an information-dependent basis (IDA) during an LC/MS experiment. In this mode, the acquisition software (Xcalibur 4.0.27, Thermo) continuously evaluates the full scan survey MS data as it collects and triggers the acquisition of MS/MS spectra depending on preselected criteria. ESI source conditions were set as following: Sheath gas flow rate as 45 Arb, Aux gas flow rate as 15Arb, Capillary temperature 320°C, Full ms resolution as 70,000, MS/MS resolution as 17,500, Collision energy as 20/40/60 eV in NCE model, Spray Voltage as 3.8 kV (positive) or − 3.1 kV (negative), respectively.

The UPLC-MS raw data (.raw) files were converted to the mzML format using ProteoWizard, and processed by R package XCMS (version 3.2). The preprocessing results generated a data matrix that consisted of the retention time (RT), mass-to-charge ratio (m/z) values, and peak intensity. OSI-SMMS (version 1.0, Dalian Chem Data Solution Information Technology Co. Ltd.) was used for peak annotation after XCMS data processing with in-house MS/MS database. Multivariate statistical analyses, including partial least squares-discriminant analysis (PLS-DA) and pairwise orthogonal projections to latent structures discriminant analyses (OPLS-DA) of different models were provided. The metabolites with variable importance projection (VIP) > 1.0 and *p* value <0.05 of Students’ unpaired t-test were considered different.

### 2.6 Targeted metabolome analysis of bile acid profiles in the liver and ileum

The bile acid profiles in the liver and ileum were subjected to targeted metabolome assays based on UHPLC–MS/MS analysis. A 25 mg aliquot of each individual sample was precisely weighed, after the addition of 1,000 μL of extract solution (pre-cooled at −40°C, acetonitrile/methanol/water, 2:2:1), the samples were vortexed for 30 s, homogenized at 35 Hz for 4 min, and sonicated for 5 min in ice-water bath. The homogenate and sonicate circle were repeated for three times, followed by incubation at −40°C for 1 h and centrifugation at 12,000 rpm and 4°C for 15 min. The resulting supernatants were taken for UHPLC–MS/MS analysis. Stock solutions were individually prepared by each standard substance dissolving or diluting to give a final concentration of 1 mg/mL. An aliquot of each of the stock solutions was transferred to a 10 mL flask to form a mixed working standard solution. For liver samples, the standard solutions (CA, cholic acid; CDCA, chenodeoxycholic acid; GCA, glycocholic acid; TCA, taurocholic acid; GCDCA, glycochenodeoxycholic acid; TCDCA, taurochenodeoxy-cholic acid) were individually prepared by dissolving or diluting each standard sub-stance to give a final concentration of 10 mmol/L. For ileal samples, a total of 69 bile acid standard solutions including the aforementioned types were provided. The UHPLC separation was carried out using an UHPLC System (Vanquish, Thermo Fisher Scientific), equipped with a Waters ACQUITY UPLC BEH C18 column (150 * 2.1 mm, 1.7 μm, Waters). The mobile phase A was 5 mmol/L ammonium acetate in water, and the mobile phase B was acetonitrile. The column temperature was set at 45°C. The autosampler temperature was set at 4°C and the injection volume was 1 μL. A Orbitrap Exploris 120 mass spectrometer (Thermo Fisher Scientific) was applied for assay development. Typical ion source parameters were: spray voltage = +3,500/−3,200 V, sheath gas (N_2_) flow rate = 40, aux gas (N_2_) flow rate = 15, sweep gas (N_2_) flow rate = 0, aux gas (N_2_) temperature = 350°C, capillary temperature = 320°C. The parallel reaction monitoring (PRM) parameters for each of the targeted analytes were optimized, by injecting the standard solutions of the individual analytes, into the API source of the mass spectrometer.

### 2.7 Statistical analyses

Data were analyzed by two-way ANOVA using SPSS 26.0 (SPSS, Inc., Chicago, IL, United States) to analyze the main effects of BBR or ETEC and their interactions for the data of mRNA expression, microbiome and targeted metabolome analyses. Duncan’s multiple comparison method was used to compare significant differences between means. Each replicate (*n* = 8) served as an experimental unit. Results are expressed as mean ± standard error of the mean (SEM). *p* < 0.05 was considered as significant difference while *p* < 0.10 indicated as trends. The comparisons of hepatic metabolites between BBR + ETEC group and ETEC group identified by non-targeted metabolome analysis were analyzed by Student’s *t-*test and were considered to be significant at the level of VIP > 0, *p* < 1 between two groups. Spearman’s correlation analysis was used to analyze the relationship between gut microbiota and bile acid or intestinal gene expression.

## 3 Results

### 3.1 The mRNA expression of intestinal barrier function and inflammatory cytokines

**Figure 1:**
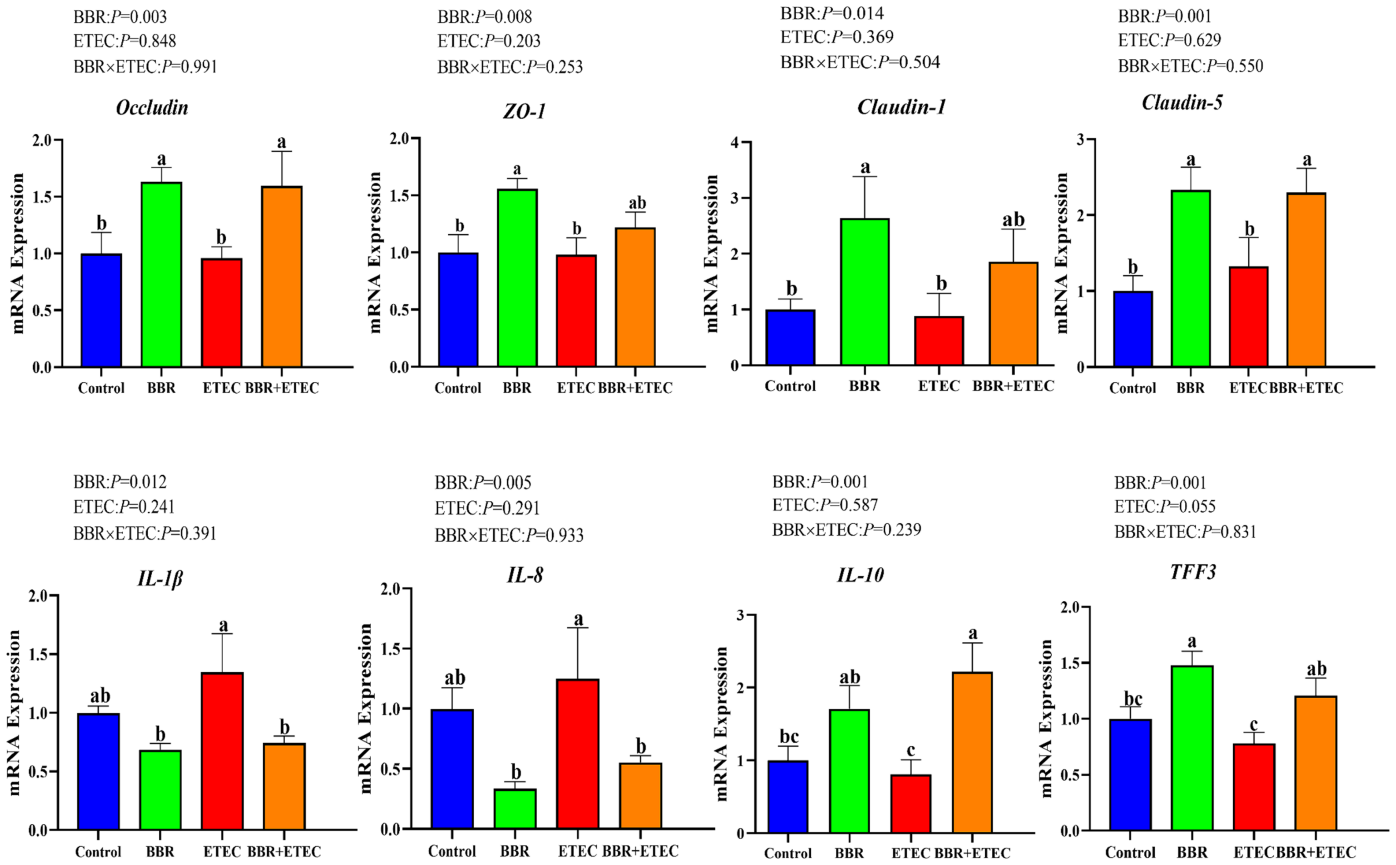
The mRNA expression of intestinal barrier and cytokines in the colon of piglets. BBR, berberine; ETEC, enterotoxigenic *Escherichia coli*; *ZO-1*, zonula occludens-1; *IL-1β*, interleukin-1β; *IL-8*, interleukin-8; *IL-10*, interleukin-10; *TFF3*, trefoil factor 3. ^a-c^Means in the columns with different superscripts differ (*p* < 0.05).

As shown in Figure 1, dietary BBR supplementation significantly unregulated the mRNA expression of *Occludin*, *Claudin-5*, trefoil factor 3 (*TFF3*) and *IL-10* in the colonic mucosa, but significantly downregulated the mRNA expression of pro-inflammatory cytokines including interleukin (*IL*)*-1β* and *IL-8* in the colonic mucosa of weaned piglets challenged with ETEC(*p* < 0.05).

### 3.2 The mRNA expression of hepatic and intestinal bile acid metabolism-related genes

**Figure 2:**
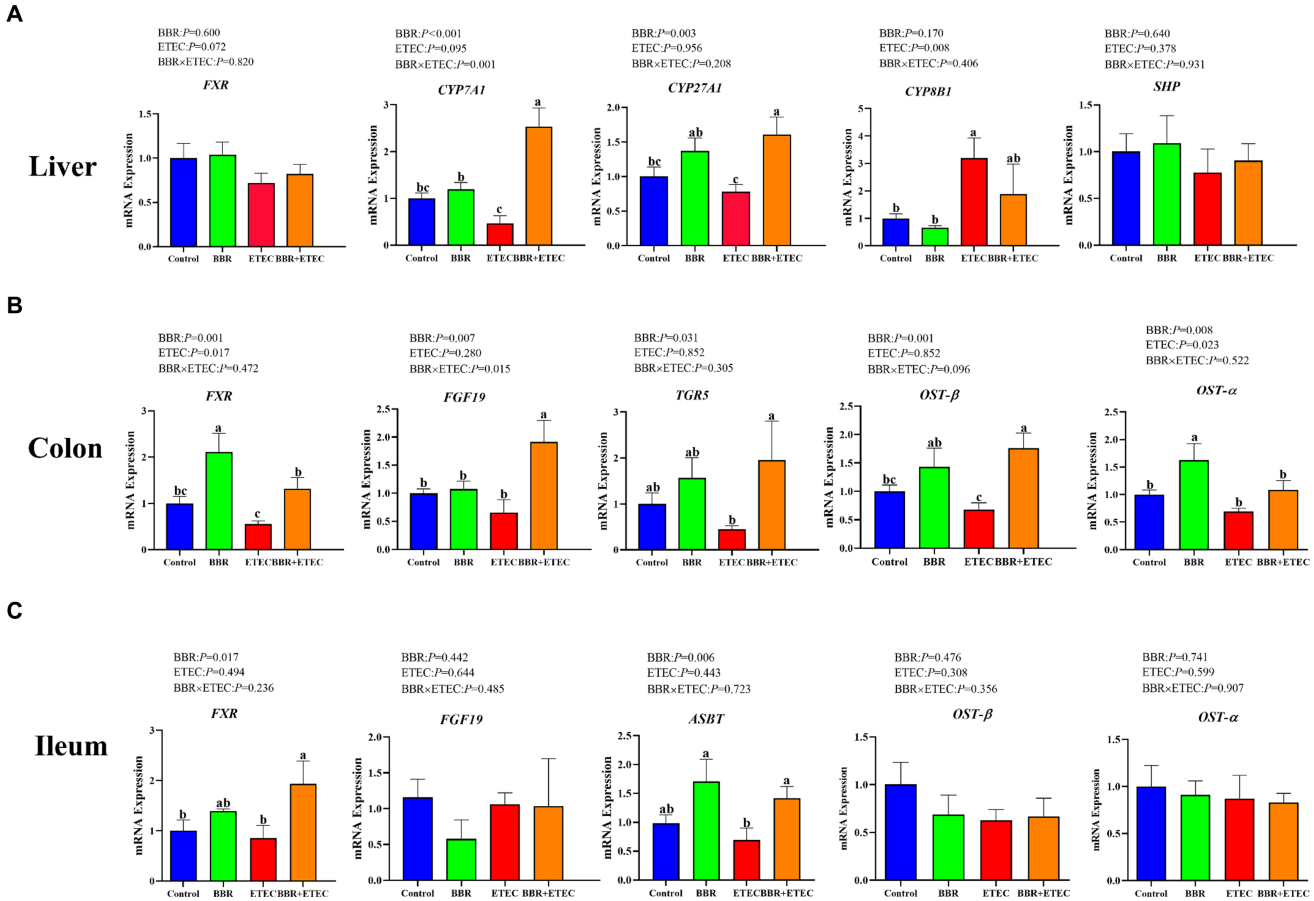
The mRNA expression of bile acid metabolism-related genes in the liver, colon, and ileum of piglets. BBR, berberine; ETEC, enterotoxigenic *Escherichia coli*. *CYP7A71*, cholesterol 7 α-hydroxylase; *CYP27A1*, sterol 27-hydroxylase; *CYP8B1*, 12a-hydroxylase; *FXR*, farnesoid X receptor; *TGR5*, takeda G protein-coupled receptor 5; *SHP*, small heterodimer partner; *FGF19*, fibroblast growth factor19; *OST-β*, organic solute transporters beta; OST-α, organic solute transporters alpha; *ASBT*, apical sodium-dependent bile acid transporter; ^a-c^Means in the columns with different superscripts differ (*p* < 0.05).

ETEC K88 challenge significantly downregulated the mRNA expression of cholesterol 7 α-hydroxylase (*CYP7A1*), and sterol 27-hydroxylase (*CYP27A1*) in the liver of weaned piglets, but significantly upregulated the mRNA expression of 12a-hydroxylase (*CYP8B1*) (*p* < 0.05) (Figure 2A). Dietary BBR addition significantly alleviated the reduction of mRNA expression of *CYP7A1* and *CYP27A1* in the livers of weaned piglets compared with the ETEC group (*p* < 0.05). However, there was no significant differences in hepatic farnesoid X receptor (*FXR*), and small heterodimer partner (*SHP*) mRNA expression among treatments (*p* > 0.05). In addition, there was a significant interaction between BBR and ETEC on hepatic *CYP7A1* mRNA expression (*p* < 0.05).

As shown in Figures 2B,C, ETEC challenge significantly downregulated the colonic *FXR*, organic solute transporters beta (*OST-β*), organic solute transporters alpha (*OST-α*), fibroblast growth factor19 (*FGF19*), takeda G protein-coupled receptor 5 (*TGR5*) and ileal *FXR* and apical sodium-dependent bile acid transporter (*ASBT*) mRNA expression in weaned piglets (*p* < 0.05). However, dietary BBR supplementation reversed the downregulated of these gene expression in weaned piglets caused by ETEC challenge (*p* < 0.05). In addition, there were no significant main effects of BBR treatment, ETEC challenge or their interactions on ileal *OST-β* and *OST-α* expression of weaned piglets (*p* > 0.05).

### 3.3 The colonic and cecal microbiota composition and diversity by 16S rRNA sequencing

**Figure 3:**
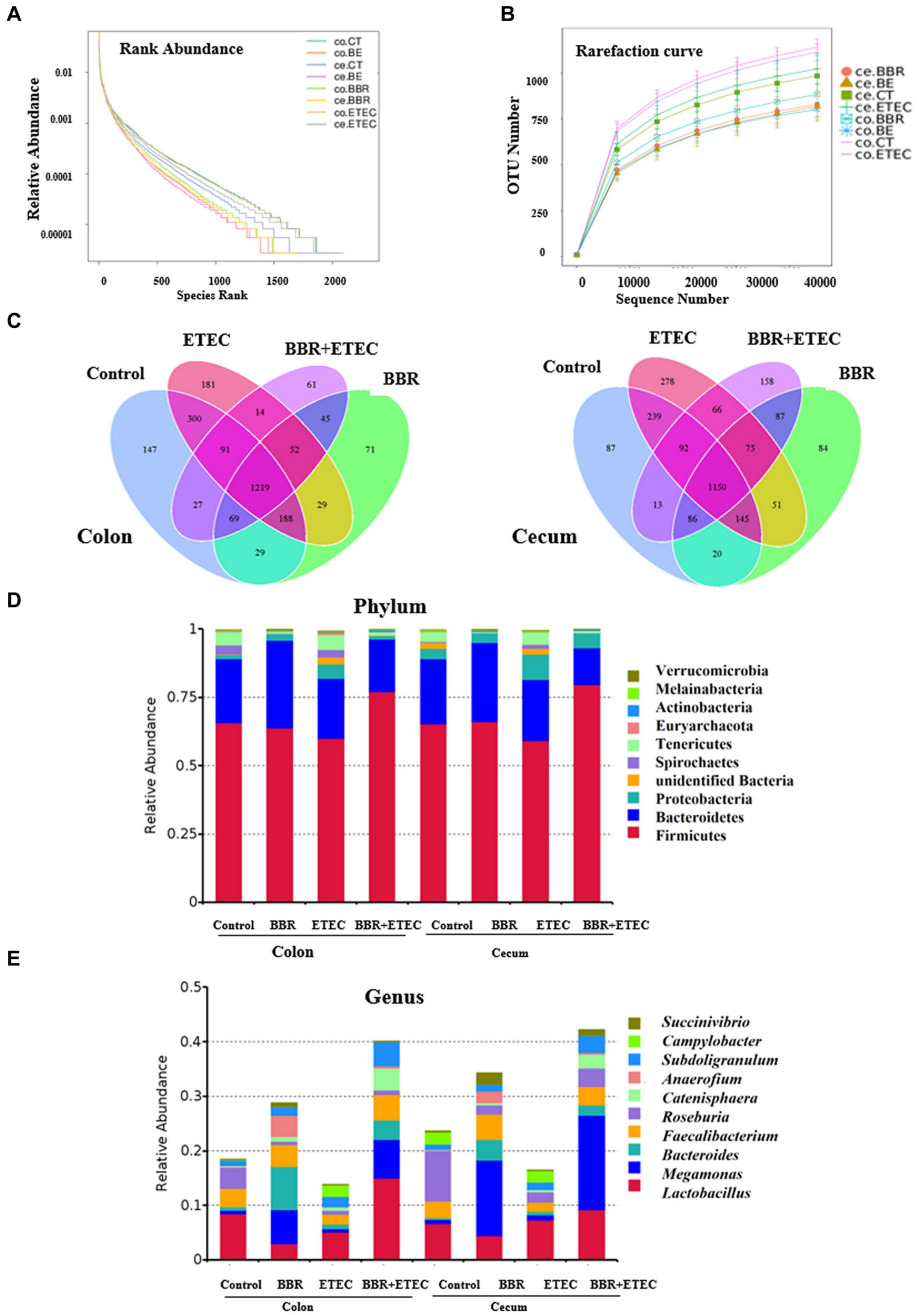
The structure of microbiota in the colonic and cecal digesta of weaned piglets in different groups. **(A)** Rank abundance. **(B)** Rarefaction curve. **(C)** Venn plot. The microbial community structure at the phylum **(D)** and genus **(E)** levels. BBR, berberine; ETEC, enterotoxigenic *Escherichia coli*.

As shown in Figures 3A,B, the rank abundance and rarefaction curve revealed sufficient OTU coverages to characterize the bacterial composition of each treatment. Specially, the Venn plot (Figure 3C) indicated that a total of 1,219 and 1,150 common OTUs were identified in the microbiota of colonic and cecal digesta in weaned piglets across the four treatments, respectively. Specially, in the colonic microbiota, there were 147 unique OTUs in the Control group, 71 in BBR group, 181 in ETEC group, and 61 in BBR + ETEC group. In the cecal microbiota, there were 87, 84, 278, and 158 unique OTUs in the Control, BBR, ETEC, and BBR + ETEC groups, respectively. These results indicated that the sequence depth was sufficient for a majority of OTUs in the colonic and cecal digesta samples.

Furthermore, the composition of top 10 most abundant phyla and genera in the colonic and cecal digesta of weaned piglets were provided. At the phylum level, the dominant phyla bacteria in the four treatment groups were Firmicute, Bacteroidetes, Proteobacteria, unidentified Bacteria, Spirochaetes, Tenericutes, Euryarchaeota, Actinobacteria, Melainabacteria, and Verrucomicrobia (Figure 3D). At the genus level, the top 10 dominant genera bacteria were *Lactobacillus*, *Megamonas*, *Bacteroides*, *Faecalibacterium*, *Roseburia*, *Catenisphaera*, *Anaerofium*, *Subdoligranulum*, *Campylobacter*, and *Succinivibrio* (Figure 3E).

**Figure 4:**
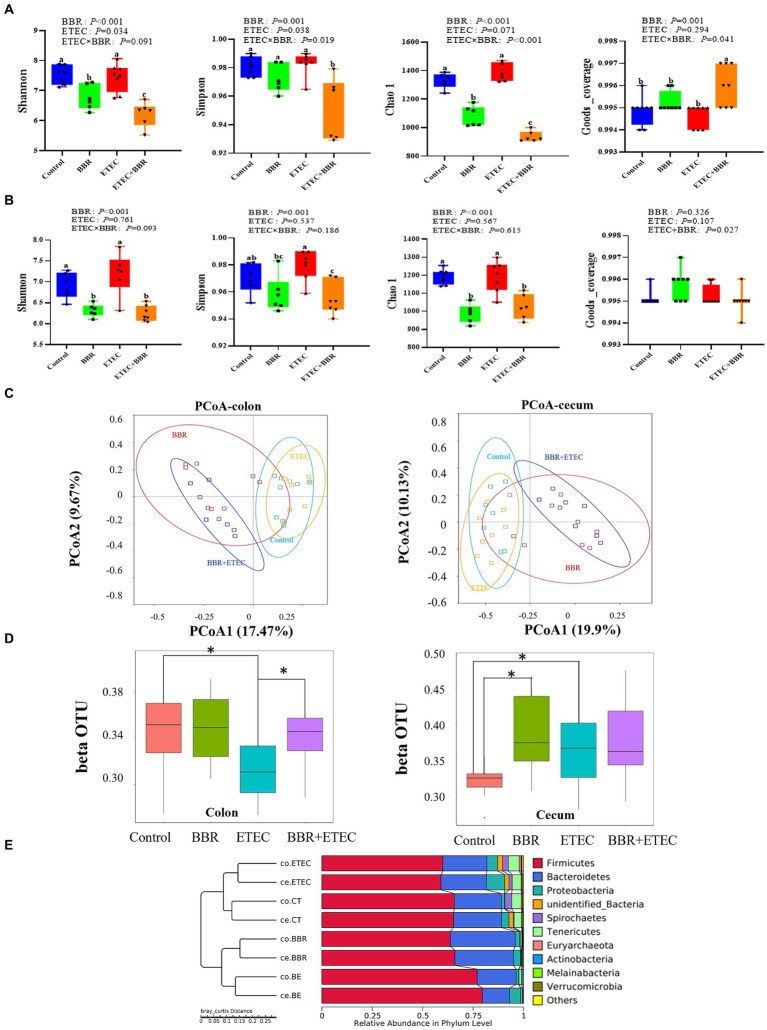
The microbial diversity in the colonic and cecal digesta of weaned piglets in different groups. **(A)** The α-diversity of colonic microbiota. **(B)** The α-diversity of ceal microbiota. **(C)** Unweighted uniFrac distance-based principal coordinate analysis (PCoA) analysis in colonic and cecal microbiota. **(D)** β-diversity index of colonic and cecal microbiota based on unweighted unfirac distance using wilcox method. * Indicated significant difference between two groups. **(E)** The UPGMA analysis based on unweighted unifrac distance. BBR, berberine; ETEC, enterotoxigenic *Escherichia coli*.

The Shannon index, Simpson index, Chao 1, and Goods_ Coverage were used to indicate the α-diversity of colonic (Figure 4A) and cecal microbiota (Figure 4B). ETEC challenge significantly increased the Shannon index, Simpson index, Chao 1, and Goods_ Coverage in the colon (Figure 4A) as well as the Shannon index, Simpson index, and Chao 1 in the cecum (Figure 4B). However, dietary BBR treatment reduced the Shannon index, Simpson index, Chao 1 in the colon (Figure 4A), but had no significant effects on these parameters in the cecum (Figure 4B). In addition, the PCoA analysis (Figure 4C) revealed that the first principal component (PCoA1) and the second principal component (PCoA2) explained 17.47 and 19.9% of the variation in the microbial diversity in the colonic digesta samples, and 9.67 and 10.13% of the variation in the microbial diversity in the cecal digesta samples, respectively. BBR group in either colon or cecum was clearly separately from Control group, while BBR + ETEC group was clearly separately from ETEC group in both colon and cecum. Moreover, the β-diversity index in the colon of ETEC group was significantly lower than the Control group, while the β-diversity index in the BBR + ETEC group was significantly higher than ETEC group (*p* < 0.05). However, the β-diversity index in the cecal microbiota both BBR group and ETEC group was significantly higher than that in the control group (Figure 4D). Moreover, The UPGMA analysis (Figure 4E) also revealed distinct differences of colonic and cecal microbiota among BBR treatment and ETEC challenge.

**Figure 5:**
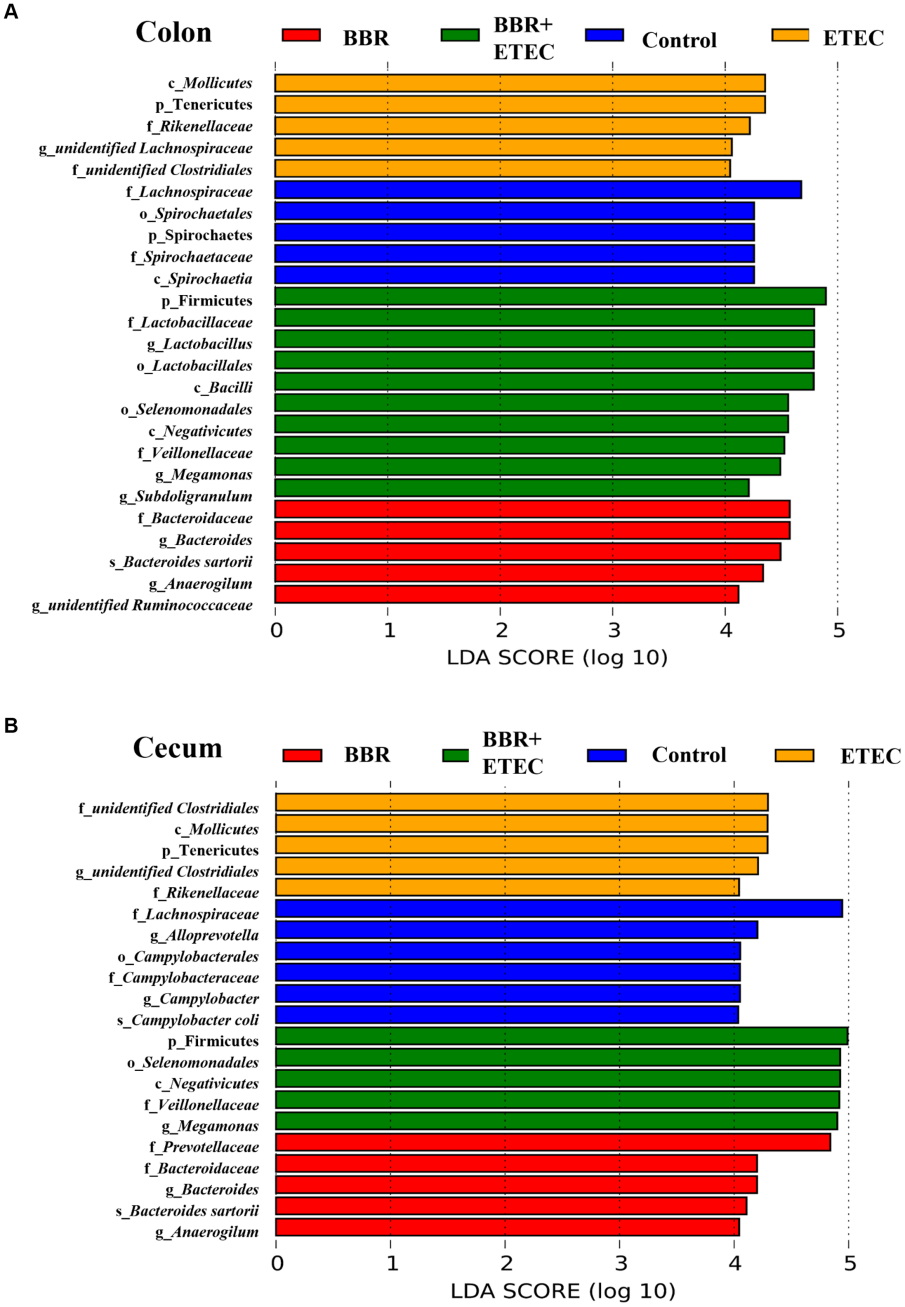
The LEfSe analysis microbiota in the colonic and cecal digesta of weaned piglets. **(A)** Colonic microbiota. **(B)** Cecal microbiota. c, class; p, phylum; f, family; o, order; g, genus; s, species; BBR, berberine; ETEC, enterotoxigenic *Escherichia coli*.

Further LEfSe analysis was used to investigate the differences in taxonomic abundance between treatments (Figure 5). There were 25 and 21 discriminative species identified among the four dietary treatments in the colonic and cecal digesta of weaned piglets, respectively. In the colon (Figure 5A), Spirochaetes (phylum), *Spirochaetia* (class), *Spirochaetales* (order), *Spirochaetaceae* (family), and *Lachnospiraceae* (family) were abundant in the colonic digesta of control group. The BBR group enriched *Bacteroidaceae* (family), *Anaerogilum* (genus), unidentified *Ruminococcaceae* (genus), *Bacteroides* (genus), *Bacteroides sartorii* (species) in the colonic digesta. Moreover, ETEC challenge alone significantly enriched 5 bacterial taxa, including Tenericutes (phylum), *Mollicutes* (class), *Rikenellaceae* (family), unidentified *Clostridiales* (family), and unidentified *Lachnospiraceae* (genus) in colonic digesta. However, Firmicutes (phylum), *Bacilli* (class), *Negativicutes* (class), *Lactobacillales* (order), *Selenomonadales* (order), *Lactobacillaceae* (family), *Veillonellaceae* (family), *Lactobacillus* (genus), *Megamonas* (genus), *Subdoligranulum* (genus) were significantly enriched in the colonic digesta of BBR + ETEC group.

In the cecum (Figure 5B), the LEfSe analysis showed that 5 bacterial taxa including *Campylobacterales* (order), *Lachnospiraceae* (family), *Campylobacteraceae* (family), *Cam-pylobacter* (genus), *Alloprevotella* (genus), and *Campylobacter coli* (species) were significantly enriched in the Control group. Dietary BBR treatment alone significantly enriched *Prevotellaceae* (family), *Bacteroidaceae* (family), *Bacteroides* (genus), *Anaerogilum* (genus), and *Bacteroides sartorii* (species). Furthermore, ETEC challenge alone significantly enriched 5 bacterial taxa including Tenericutes (phylum), *Mollicutes* (class), unidentified *Clostridiales* (family), unidentified *Clostridiales* (genus), and *Rikenellaceae* (family), while another 5 bacterial taxa including Firmicutes (phylum), *Negativicutes* (class), *Selenomonadales* (order), *Veillonellaceae* (family), and *Megamonas* (genus) were significantly enriched in BBR + ETEC group.

**Figure 6:**
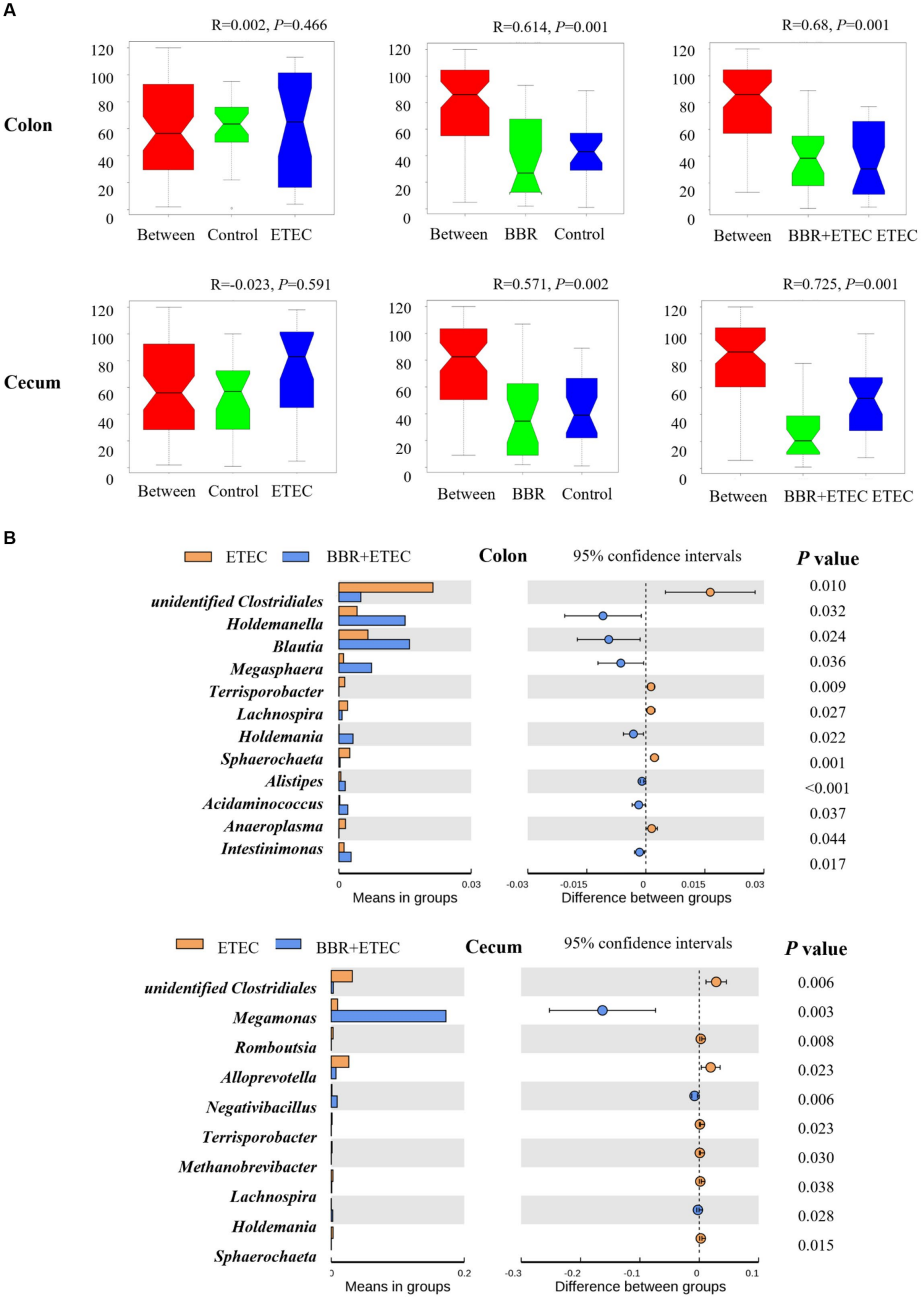
The analysis of similarities (Anosim) and *T*-test analysis for the significant changes of differential microbiota at genus level. **(A)** Anosim test. **(B)** *T*-test. BBR, berberine; ETEC, enterotoxigenic *Escherichia coli*.

The ANOSIM analysis (Figure 6A) showed that the microbial structures of the colonic digesta samples (*R* = 0.641, *p* = 0.001) as well as cecal digesta samples (*R* = 0.571, *p* = 0.002) were significantly different between the Control group and BBR group. Moreover, there were significant differences between the BBR + ETEC and ETEC treatments in both colonic (*R* = 0.68, *p* = 0.001) and cecal microbiota (*R* = 0.725, *p* = 0.001) in the colon and cecum digesta of weaned piglets. Further *T*-test results for differential bacteria comparisons among groups at the genus level were shown in Figure 6B. In the colonic digesta, when compared to the ETEC group, the relative abundances of *Holdemanella*, *Blautia*, *Megasphaera*, *Holdemania*, *Alistipes*, *Acidaminococcus*, and *Intestinimonas* were significantly increased in BBR + ETEC groups (*p* < 0.05), while the relative abundance of unidentified *Clostridiales*, *Terrisporobacter*, *Lachnospira*, *Sphaerochaeta*, *Anaeroplasma* were decreased in BBE + ETEC groups (*p* < 0.05). Moreover, the relative abundance of *Megasphaera* and *Negativibacillus* were significantly increased in BBR + ETEC groups (*p* < 0.05), but those of unidentified *Clostridiales*, *Romboutsia*, *Alloprevotella*, *Terrisporobacter*, *Methanobrevibacter*, *Lachnospira*, and *Sphaerochaeta* were decreased in BBR + ETEC group compared to the ETEC group (*p* < 0.05).

### 3.4 Altered bile acid profiles in the gut-liver axis of weaned piglets fed diets with BBR supplementation via non-targeted and targeted metabolome analyses

**Figure 7:**
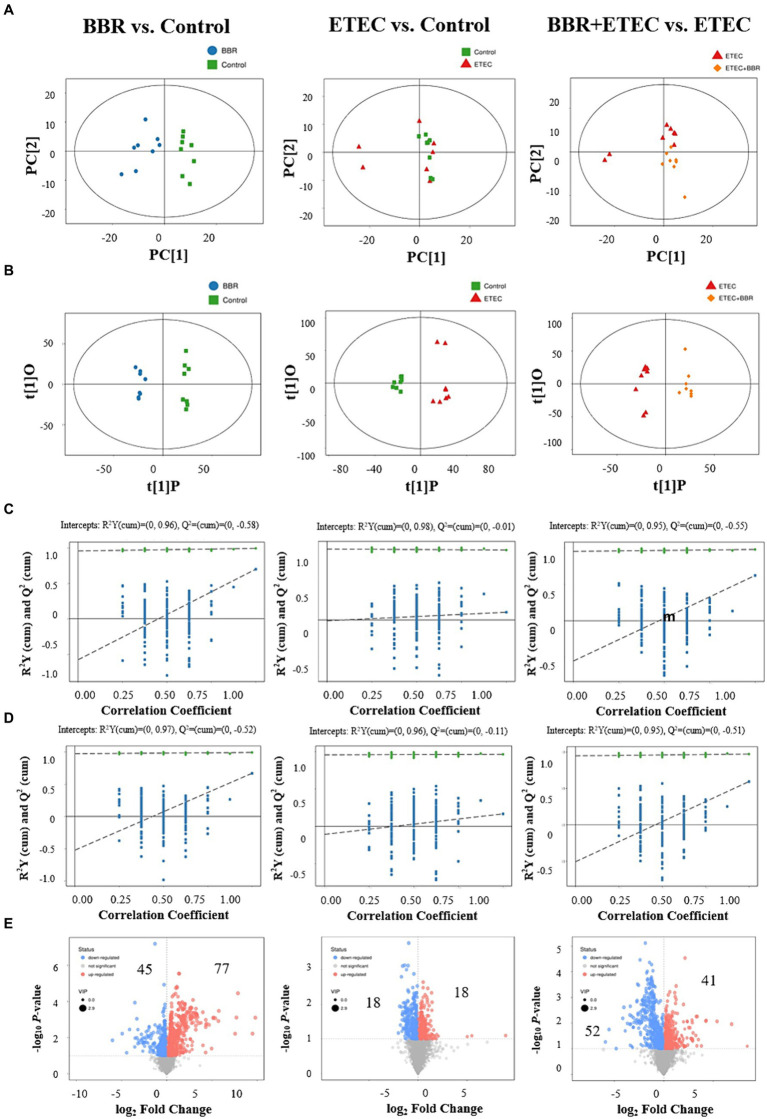
Multivariate statistical analysis of untargeted metabolome data of liver. The OPLS-DA Score plot **(A)** and permutation plot **(B)** from liver samples in BBR (blue dots), Control (green dots), ETEC (red dots), BBR + ETEC (orange dots) groups. Permutation validation of OPLS-DA models on positive **(C)** and negative **(D)** ion modes, respectively. Blue dots and green dots represented *Q*^2^ and *R*^2^Y, respectively. **(E)** The volcano plots for BBR vs. Control, ETEC vs. Control, and BBR + ETEC vs. ETEC, with blue and red dots representing the upregulated and downregulated metabolites in the livers, respectively. BBR, berberine; ETEC, enterotoxigenic *Escherichia coli*.

The score-plot of the PLS-DA models from liver metabolome of weaned piglets revealed that the hepatic metabolome of the control group could be clearly distinguished from that of BBR group and ETEC group (Figures 7A,B). Moreover, the hepatic metabolome of the BBR + ETEC group was also clearly separated from that of ETEC group (Figures 7A,B). Results from the permutation test of OPLS-DA analyses also showed the groups within the models were reliable and significantly different (Figures 7C,D) (BBR vs. Control, positive ion mode, *R*^2^*Y* = 0.96, *Q*^2^ = –0.58; negative ion mode, *R*^2^*Y* = 0.97, *Q*^2^ = −0.52; BBR vs. Control, positive ion mode, *R*^2^*Y* = 0.98, *Q*^2^ = −0.01; negative ion mode, *R*^2^*Y* = 0.96, *Q*^2^ = −0.11; BBR + ETEC vs. ETEC, positive ion mode, *R*^2^*Y* = 0.95, *Q*^2^ = –0.05; negative ion mode, *R*^2^*Y* = 0.96, *Q*^2^ = −0.55). These parameters for evaluating the predictive ability and fitting level of the models resulting from internal validation suggested that the OPLS-DA models possessed a satisfactory fit with good predictive power. These results indicated that piglets receiving BBR-supplemented diets showed distinctive metabolic profiles in the livers compared with piglets challenged with or without ETEC infection.

Furthermore, according to the OPLS-DA Score-plot and the volcano plot (Figure 7E), we detected 122 (77 upregulated and 45 downregulated), 36 (18 up-regulated and 18 down-regulated), and 93 (41 up-regulated and 52 down-regulated) differential metabolites between BBR vs. Control group, ETEC vs. Control group, and BBR + ETEC vs. ETEC group, respectively. Specially, the differential metabolites between BBR + ETEC and ETEC group based on the criteria of VIP > 1, *p* < 0.05, and fold-change ratio ≥ 2 or ≤ 0.5 were shown in Table 2. Importantly, the concentrations of TCDCA and taurine were significantly increased by dietary supplementation with BBR in ETEC-challenged piglets (Table 2).

**Table 2:** The hepatic differential metabolites between BBR + ETEC group and ETEC group of piglets identified by non-targeted metabolomic analysis.

**Differential metabolites**	**RT**	**VIP**	***p*-value**	**Fold change**
ADPribose 2′-phosphate	368.38	2.32	0.011	10.71↑
*Trans*-Zeatin riboside diphosphate	408.76	2.75	0.005	10.65↑
Gallagic acid	449.66	2.44	0.007	6.54↑
CDP-ribitol	395.12	2.56	0.004	6.10↑
UDP-L-Ara4N	378.82	2.57	0.005	6.08↑
3’-Phosphoadenylyl sulfate	350.17	1.86	0.008	4.36↑
Neosurugatoxin	500.21	2.26	0.003	4.28↑
DTDP-alpha-D-glucose (2-)	392.18	1.80	0.016	3.92↑
3-[3,4-dihydroxy-5-(3,4,5-trihydroxybenzoyloxy) benzoyloxy]-2,4,5-trihydroxybenzoic acid	382.28	1.23	0.028	3.22↑
UDP-4-dehydro-6-deoxy-D-glucose	401.00	2.39	0.004	3.11↑
Octacosyl triacontanoate	493.27	2.62	0.001	2.93↑
Tellimagrandin I	506.90	2.31	0.021	2.84↑
Pyrazolate	426.67	2.52	0.001	2.80↑
CDP-ribitol	353.39	1.68	0.039	2.53↑
Chondroitin 4-sulfate	486.58	2.26	0.001	2.48↑
3-{3-[3,5-dihydroxy-4-(sulfooxy) benzoyloxy]-4,5-dihydroxybenzoyloxy}-4,5-dihydroxybenzoic acid	442.75	1.76	0.021	2.46↑
Taurochenodesoxycholic acid	293.67	2.10	0.000	2.39↑
2,3,4,7,8,9,15,21,22,23,28-undecahydroxy-14-(hydroxymethyl)-12,27,31,34-tetraoxo-13,25,32,35-tetraoxaoctacyclo pentatriaconta-1,3,5(30),6,8,10,19,21,23,28-decaene-20-carboxylic acid	495.42	2.18	0.002	2.30↑
GDP-4-amino-4,6-dideoxy-alpha-D-mannose	412.24	1.74	0.016	2.28↑
Phosphatidylglyceride 20:4–22:6	489.23	2.62	0.001	2.26↑
1,8,16,17,18,21,22,23,34,35,39,39-dodecahydroxy-6,9,12,27,30,40-hexaoxaoctacyclo tetraconta-3,14,16,18,20,22,24,32,34,36-decaene-2,5,13,26,31-pentone	510.90	2.59	0.001	2.20↑
DTDP-alpha-D-glucose (2-)	406.62	1.52	0.023	2.19↑
Rebeccamycin	441.11	1.60	0.026	2.18↑
Molybdenum cofactor (sulfide)	431.22	2.18	0.007	2.09↑
6-Hydroxy flavin adenine dinucleotide	510.65	2.40	0.002	2.03↑
3,4,5-trihydroxy-6-{[7,8,8,13,22-pentahydroxy-19-(hydroxymethyl)-3,6,16-trioxo-21-(3,4,5-trihydroxybenzoyloxy)-2,17,20,23-tetraoxapentacycl tricosa-4,10,12,14-tetraen-12-yl] oxy} oxane-2-carboxylic acid	506.91	1.74	0.023	2.03↑
Taurine	30.90	2.30	0.001	2.01↑
2-O-[2-O-(alpha-D-Mannopyranosyl)-alpha-D-glucopyranosyl]-3-phospho-D-glycerate	485.50	2.07	0.003	1.98↑
Molybdopterin	401.00	2.60	0.001	1.98↑
Opthalmic acid	36.56	2.42	0.001	0.45↓
CMP-*N*-glycoloylneuramite	470.84	1.73	0.036	0.44↓
Cyanotriphenylborate	36.03	2.33	0.002	0.44↓
6-[5-(3-{[3,4-dihydroxy-5-(hydroxymethyl) oxolan-2-yl] oxy}-5,7-dihydroxy-4-oxo-4H-chromen-2-yl)-2-hydroxyphenoxy]-3,4,5-trihydroxyoxane-2-carboxylic acid	470.80	1.61	0.043	0.44↓
Uracil mustard	191.90	1.65	0.031	0.44↓
Salicyluric beta-D-glucuronide	490.32	1.63	0.010	0.43↓
5-(acetyloxy)-14-hydroxy-9-oxo-8,17-dioxatetracyclo heptadeca-1(10),2(7),3,5,11,13,15-heptaen-13-yl acetate	490.32	1.57	0.040	0.42↓
Ioxynil octanoate	149.95	1.26	0.034	0.28↓
Fipronil	193.21	2.09	0.040	0.14↓
4’-Hydroxydiclofenac	308.67	2.01	0.008	0.14↓
2,2′,5,5’-Tetrachlorobenzidine	172.46	2.53	0.008	0.05↓

**Figure 8:**
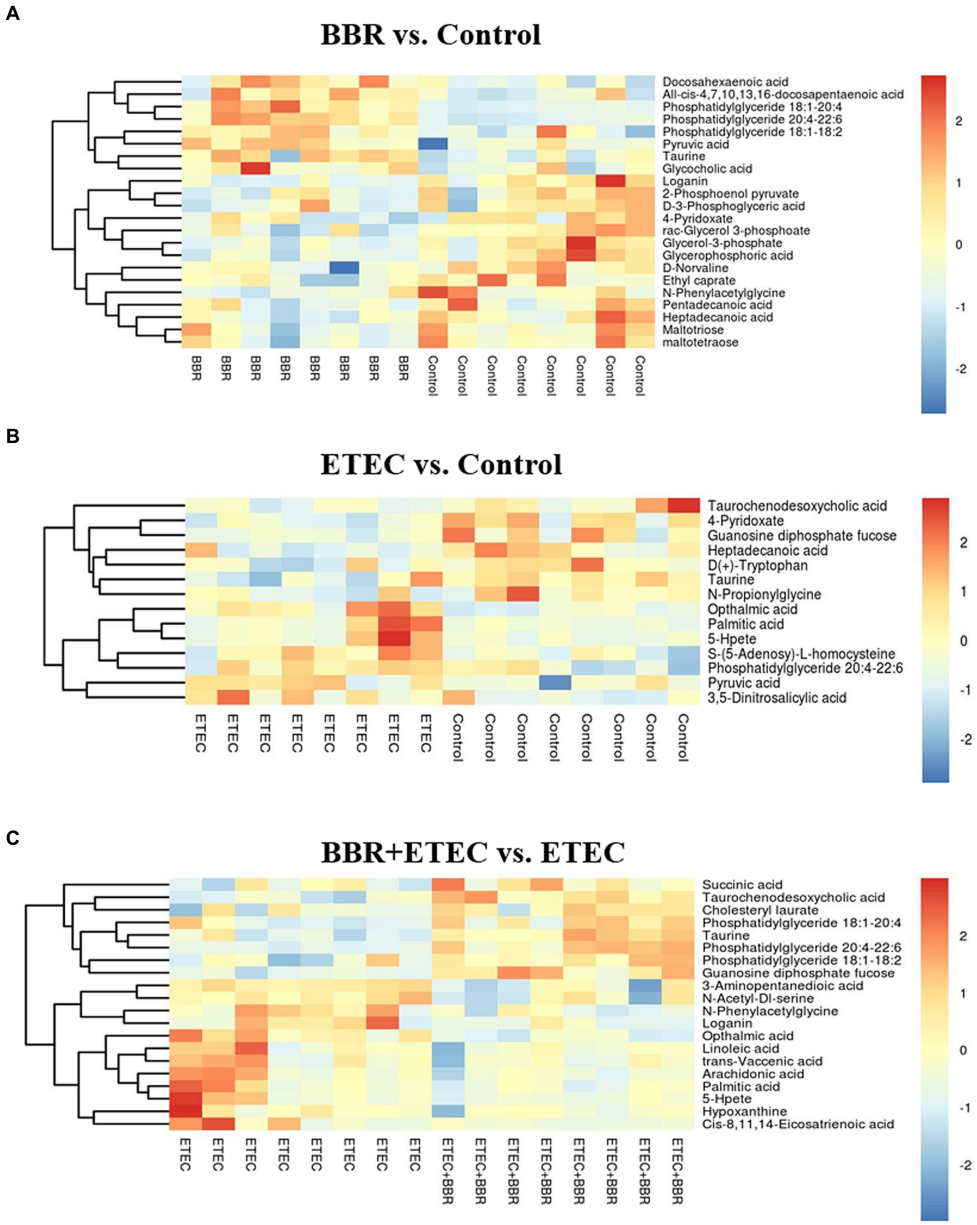
The differential metabolites in the livers of weaned piglets from non-targeted metabolome analysis for BBR vs. Control **(A)**, ETEC vs. Control **(B)**, and BBR + ETEC vs. ETEC **(C)**, respectively. The non-targeted metabolome data were normalized by logarithmic and Euclidean distance and produced a heat map. BBR, berberine; ETEC, enterotoxigenic *Escherichia coli*.

The relevant significant pathways were enriched for those selected metabolites based on *p*-value (<0.05), of which the primary bile acid biosynthesis was abundantly enriched when comparing BBR and Control, ETEC and Control, as well as BBR + ETEC and ETEC groups (Figure 8). Specifically, the differential metabolites between BBR + ETEC and ETEC groups (Table 3), were mainly enriched the following 16 pathways including: (1) Purine metabolism (Hypoxanthine), (2) Primary bile acid biosynthesis (Taurine; Taurochenodesoxycholic acid), (3) Biosynthesis of unsaturated fatty acids (Palmitic acid; Linoleic acid; Arachidonic acid; 8,11,14-Eicosatrienoic acid), (4) Fatty acid metabolism (Palmitic acid), (5) Fatty acid biosynthesis (Palmitic acid), (6) Amino sugar and nucleotide sugar metabolism (GDP-L-fucose), (7) Arachidonic acid metabolism (Arachidonic acid; Hydroperoxyeicosatetraenoic acid), (8) Steroid biosynthesis (Palmitic acid), (9) Fatty acid elongation in mitochondria (Palmitic acid), (10) Alanine, aspartate and glutamate metabolism (Succinic acid), (11) Propanoate metabolism (Succinic acid), (12) Butanoate metabolism (Succinic acid), (13) Citrate cycle (TCA cycle, Succinic acid), (14) Fructose and mannose metabolism (GDP-L-fucose), (15) Taurine and hypotaurine metabolism (Taurine), (16) Linoleic acid metabolism (Linoleic acid).

**Table 3:** The metabolic pathway enrichment analysis of the differential metabolites in the livers of weaned piglets fed diets supplemented with berberine followed by enterotoxigenic *Escherichia coli* K88 challenge.

**Pathway**	**Total**	**Hits**	**Raw P**	**FDR**	**Impact**	**Hits compounds**
**BBR + ETEC vs. ETEC**						
Purine metabolism	68	1	0.51	1	0.01	Hypoxanthine
Primary bile acid biosynthesis	46	2	0.08	1	0.06	Taurine; Taurochenodesoxycholic acid
Biosynthesis of unsaturated fatty acids	42	4	0.00	0.04	0.00	Palmitic acid; Linoleic acid; Arachidonic acid; 8,11,14-Eicosatrienoic acid
Fatty acid metabolism	39	1	0.33	1	0.00	Palmitic acid
Fatty acid biosynthesis	38	1	0.32	1	0.00	Palmitic acid
Amino sugar and nucleotide sugar metabolism	37	1	0.32	1	0.01	GDP-L-fucose
Arachidonic acid metabolism	36	2	0.05	1	0.44	Arachidonic acid; Hydroperoxyeicosatetraenoic acid
Steroid biosynthesis	35	1	0.30	1	0.00	Palmitic acid
Fatty acid elongation in mitochondria	27	1	0.24	1	0.00	Palmitic acid
Alanine, aspartate and glutamate metabolism	23	1	0.21	1	0.00	Succinic acid
Propanoate metabolism	20	1	0.18	1	0.00	Succinic acid
Butanoate metabolism	20	1	0.18	1	0.00	Succinic acid
Citrate cycle (TCA cycle)	20	1	0.18	1	0.03	Succinic acid
Fructose and mannose metabolism	19	1	0.18	1	0.02	GDP-L-fucose
Taurine and hypotaurine metabolism	7	1	0.07	1	0.75	Taurine
Linoleic acid metabolism	5	1	0.05	1	1.00	Linoleic acid

**Figure 9:**
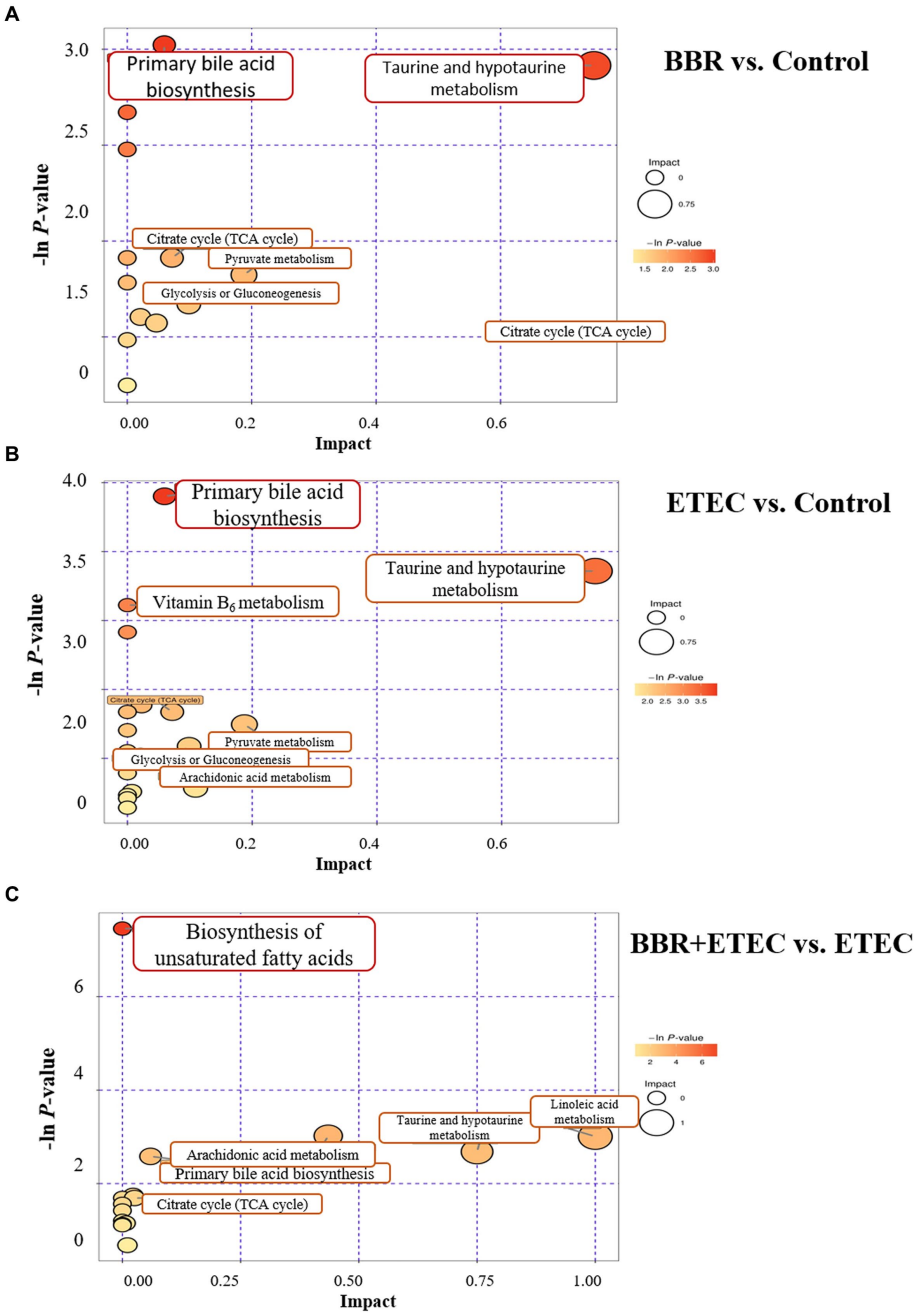
The KEGG pathway enrichment for differential metabolites from non-targeted metabolome analysis for BBR vs. Control **(A)**, ETEC vs. Control **(B)**, and BBR + ETEC vs. ETEC **(C)**, respectively. BBR, berberine; ETEC, enterotoxigenic *Escherichia coli*.

The heatmap analysis of hepatic non-targeted metabolome data revealed that the concentrations of hepatic taurine and TCDCA related to primary bile acid biosynthesis were both upregulated by dietary BBR supplementation in weaned piglets challenged with ETEC (*p* < 0.05) (Figure 9). Additionally, further hepatic targeted metabolome study also confirmed that the TCDCA concentration in the liver of BBR + ETEC group was increased by dietary BBR supplementation when compared to that in ETEC group (*p* < 0.01). The ETEC group had lower concentrations of TCDCA and conjugated bile acids in the liver of weaned piglets in contrast to the Control group and BBR group, respectively (Table 4). Moreover, the CA concentration was downregulated by dietary BBR supplementation when compared to that in ETEC group (*p* < 0.01). In addition, there were no significant differences in the concentrations of unconjugated bile acids in the liver of piglets among groups (*p* > 0.05).

**Table 4:** Quantitative results for the bile acid metabolites in the liver and ileum of weaned piglets by targeted metabolomic analysis.

**Item**	**Group**				**SEM**	***p-value***		
	**Control**	**BBR**	**ETEC**	**BBR + ETEC**		**BBR**	**ETEC**	BBR*ETEC
**Liver (nmol/g)**								
CA	0.07^b^	0.03^c^	0.12^a^	0.02^c^	0.01	<0.001	0.058	0.007
CDCA	0.26	0.21	0.24	0.21	0.02	0.231	0.845	0.815
GCA*	1.37^a^	1.35^a^	1.52^a^	0.78^b^	0.10	0.049	0.249	0.062
TCA*	0.51^ab^	0.65^a^	0.30^b^	0.57^a^	0.05	0.016	0.101	0.644
GCDCA*	121.54	136.67	107.65	108.53	5.45	0.452	0.058	0.502
TCDCA*	30.26^b^	44.40^a^	17.48^c^	37.91^ab^	2.65	<0.001	0.012	0.377
Unconjugated bile acids	0.36	0.27	0.38	0.27	0.02	0.050	0.651	0.307
Conjugated bile acids	157.23^ab^	190.34^a^	130.02^b^	148.84^ab^	7.60	0.101	0.036	0.636
**Ileum (nmol/kg)**								
CA	7600.92	3163.48	14307.58	1310.95	2562.85	0.103	0.628	0.399
CDCA	91817.93	192794.44	106371.06	41246.44	28256.21	0.748	0.239	0.162
TCDCA*	25524.87	274031.03	262249.98	109077.23	63556.91	0.722	0.788	0.159
TCA*	463.07	3059.94	4735.34	4002.64	810.51	0.557	0.120	0.304
GCDCA*	4306.85^b^	525699.29^a^	70653.58^b^	193485.36^ab^	80739.46	0.033	0.320	0.151
GCA*	242.67	14636.01	12636.79	5685.83	9693.39	0.492	0.748	0.073
HCA	411807.91^ab^	158302.44^bc^	507868.64^a^	59569.39^c^	69947.84	0.009	0.990	0.366
DCA	968.22^b^	479.53^b^	4315.26^a^	211.36^b^	576.25	0.018	0.084	0.049
LCA	1331.20	263.39	1405.25	602.47	241.19	0.071	0.662	0.779
UDCA	879.97	12796.68	1891.17	700.19	2547.16	0.283	0.268	0.196
HDCA	509101.47^a^	22653.82^b^	238343.06^ab^	67990.01^b^	76265.85	0.023	0.365	0.215
GHDCA*	36267.17	960358.38	1373492.24	482815.37	244176.87	0.972	0.369	0.073
THDCA*	82040.10	302339.63	495612.49	339954.79	77017.75	0.832	0.164	0.238
7-KDCA	1447.78^ab^	180.41^b^	3457.57^a^	220.37 ^b^	547.64	0.033	0.274	0.292
β-MCA	606.40^a^	85.03^b^	207.86^b^	13.71^b^	82.36	0.009	0.056	0.158
GUDCA-3S*	48.15^c^	157.35^a^	59.97^bc^	132.13^ab^	17.13	0.005	0.792	0.492
NCA	8.24^b^	22.69^ab^	40.38^a^	10.21^b^	5.23	0.361	0.261	0.027
Primary bile acids	439906.81	3235062.83	3218646.80	1188070.52	719936.12	0.796	0.804	0.120
Secondary bile acids	710851.75	1617040.21	2443784.72	714740.79	336763.32	0.524	0.521	0.058
Unconjugated bile acids	1010985.60^a^	366334.04^b^	952221.30^ab^	143807.20^b^	164497.39	0.005	0.274	0.787
Conjugated bile acids	139840.25	4485789.41	4710259.82	1759073.58	934816.92	0.701	0.613	0.062
Total bile acids	1150825.85	4852123.45	5662481.13	1902880.77	983713.90	0.988	0.689	0.074

CA, cholic acid; CDCA, chenodeoxycholic acid; GCA, glycocholic acid; TCA, taurocholic acid; GCDCA, glycochenodeoxycholic acid; TCDCA, taurochenodeoxy cholic acid; HCA, hyocholic acid; DCA, deoxycholic acid; UDCA, ursodeoxycholic acid; HDCA, hyodeoxycholic acid; GHDCA, glycohyodeoxycholic acid; LCA, lithocholic acid; THDCA, taurohyodeoxycholic acid; 7-KDCA, 7-ketodeoxycholic acid; β-MCA, β-muricholic acid; GUDCA-3S, glycoursodeoxycholic acid-3-sulfate; NCA, nor cholic acid. Result for mean ± standard error. ^a,b^ Indicate a significant difference at *p* < 0.05 (*n* = 8), * indicate conjugated bile acids.

Considering the enterohepatic circulation, we then conducted the targeted metabolome analysis of ileal contents to investigate the impact of BBR on intestinal bile acids profile in ETEC-challenged piglets (Table 4). The present findings revealed that dietary BBR supplementation significantly reduced the elevated hyocholic acid (HCA), deoxycholic acid (DCA), 7-ketodeoxycholic acid (7-KDCA) and norcholic acid (NCA) concentrations in the ileum of weaned piglets induced by ETEC challenge (*p* < 0.05). Furthermore, when compared with the Control group, the concentrations of hyodeoxycholic acid (HDCA), β-muricholic acid (β-MCA), and unconjugated bile acids in the ileum of weaned piglets were significantly decreased in the BBR group, while GCDCA and glycoursodeoxycholic acid-3-sulfate (GUDCA-3S) concentrations were significantly increased in the BBR group (*p* < 0.05). However, there were no significant differences in concentration of primary bile acids, secondary bile acids, conjugated bile acids, and total bile acids in the ileum contents of weaned piglets among groups (*p* > 0.05).

### 3.5 Spearman correlation with gut microbiota and bile acids or host gene expression

**Figure 10:**
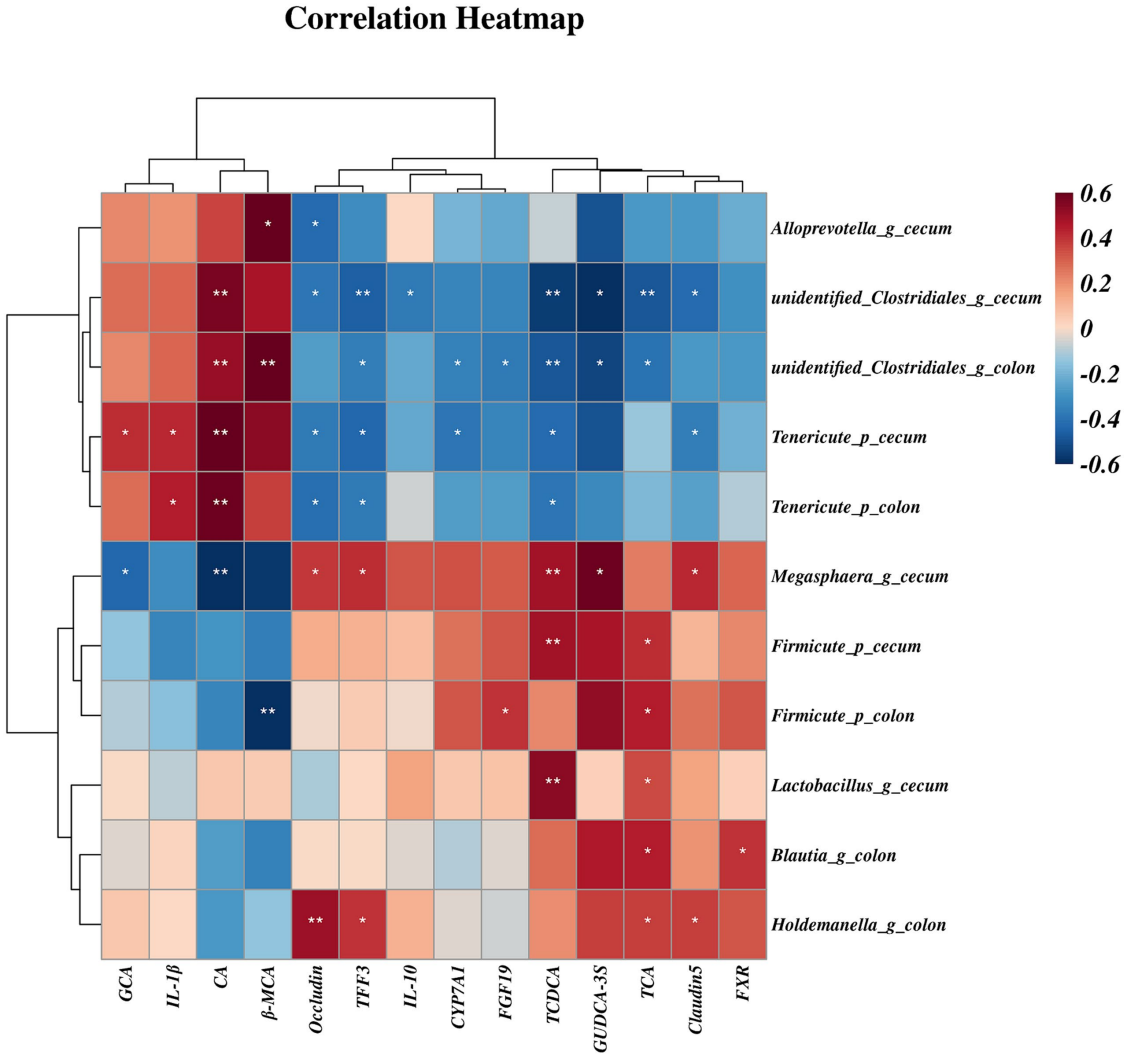
The spearman correlation between gut microbiota and bile acid concentrations or gene expression. Spearman correlation coefficients (from −0.6 to 0.6) are represented by color ranging from red (positive correlation) to blue (negative correlation). * and ** Indicate statistically significant difference at *p* < 0.05 and *p* < 0.01, respectively. CA, cholic acid; GCA, glycocholic acid; TCA, taurocholic acid; TCDCA, taurochenodeoxy cholic acid; β-MCA, β-muricholic acid; GUDCA-3S, glycoursodeoxycholic acid-3-sulfate; *CYP7A71*, cholesterol 7α-hydroxylase; *FXR*, farnesoid X receptor; *FGF19*, fibroblast growth factor19; *TFF3*, trefoil factor 3; *IL-1β*, interleukin-1β; *IL-10*, interleukin-10.

**Figure 11:**
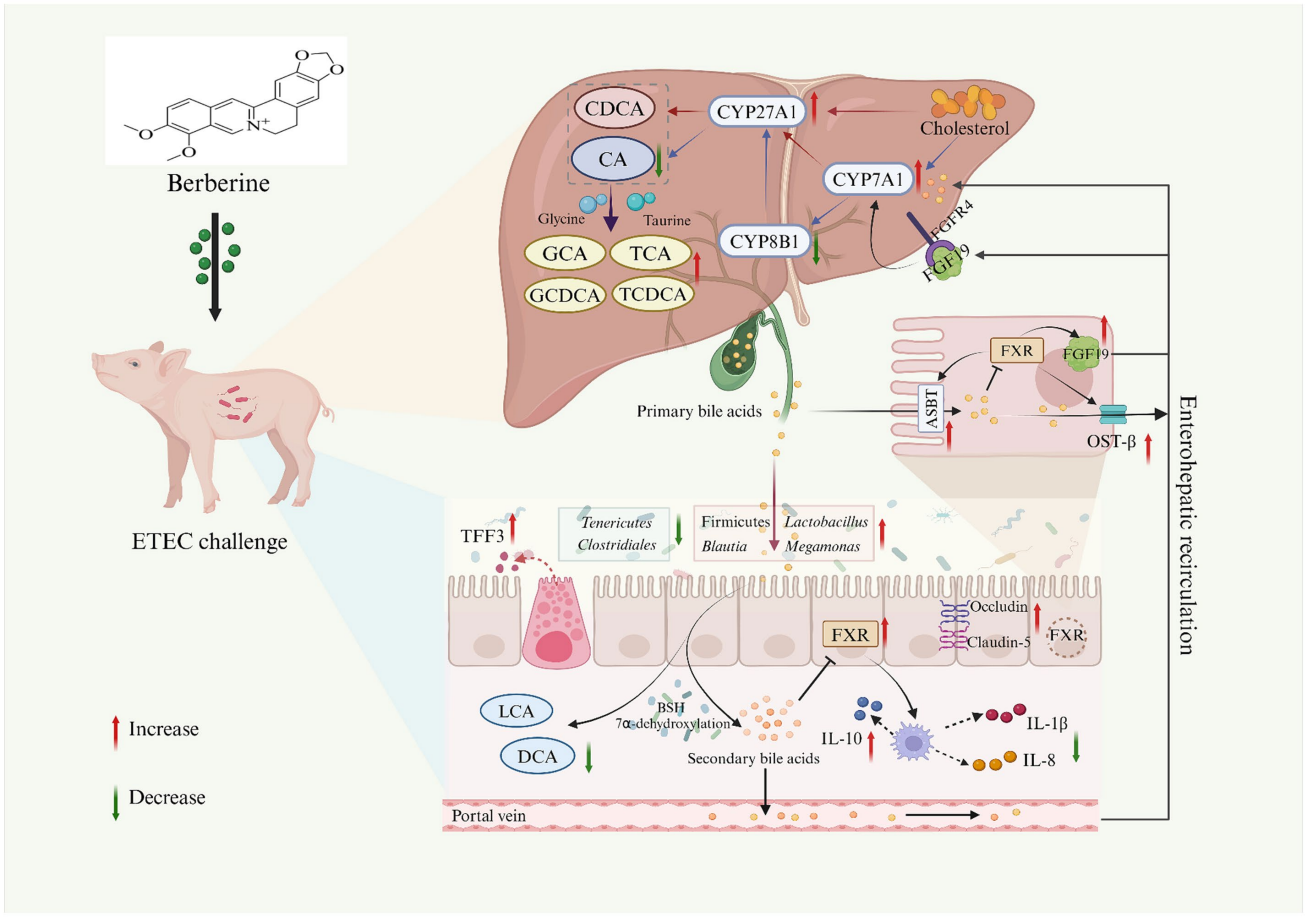
The potential mechanism of dietary BBR supplementation on the gut microbiota-bile acid-FXR axis in ETEC-challenged weaned piglets. Created with BioRender.com. CA, cholic acid; CDCA, chenodeoxycholic acid; GCA, glycocholic acid; TCA, taurocholic acid; GCDCA, glycochenodeoxycholic acid; TCDCA, taurochenodeoxy cholic acid; DCA, deoxycholic acid; LCA, lithocholic acid; CYP7A71, cholesterol 7α-hydroxylase; CYP27A1, sterol 27-hydroxylase; CYP8B1, 12a-hydroxylase; FXR, farnesoid X receptor; FGF19, fibroblast growth factor19; ASBT, apical sodium-dependent bile acid transporter; TFF3, trefoil factor 3; IL-1β, interleukin-1β; IL-8, interleukin-8; IL-10, interleukin-10.

Further Spearman analyses (Figure 10) showed that mRNA expression of *Occludin* was negatively associated with relative abundances of cecal *Alloprevotella* (genus), unidentified *Clostridiales* (genus), Tenericutes (phylum) and colonic Tenericutes (phylum). The mRNA expression of *Claudin-5* and ileal β-MCA concentration was negatively correlated with the relative abundances of cecal unidentified *Clostridiales* (genus) and Tenericutes (phylum). However, the relative abundances of cecal *Megamonas* (genus) and colonic *Holdemanella* (genus) were both positively correlated with mRNA expression of *Occludin* and *Claudin-5* (*p* < 0.05). Moreover, the relative abundance of Tenericutes (phylum) of the colon and cecum was positively correlated with *IL-1β* (*p* < 0.05). The relative abundances of colonic and cecal unidentified *Clostridiales* (genus) and Tenericutes (phylum) were positively correlated with hepatic CA concentration, but were negatively correlated with mRNA expression of *TFF3* and hepatic TCDCA concentration (*p* < 0.05). The relative abundances of cecal *Lactobacillus* were also positively correlated with mRNA expression of the hepatic concentrations of TCA and TCDCA (*p* < 0.05). Additionally, *Megamonas* (genus) and Firmicute (phylum) of the cecum were positively correlated with hepatic TCDCA concentration, and *Megamonas* (genus) was negatively correlated with hepatic CA concentration, but positively correlated with mRNA expression of *TFF3* (*p* < 0.05). The ileal GUDCA-3S concentration was positively correlated with the relative abundance of *Megamonas* (genus), and negatively correlated with unidentified *Clostridiales* (genus) (*p* < 0.05). The hepatic GCA concentration was positively correlated with the relative abundance of Tenericutes (phylum), and negatively correlated with *Megamonas* (genus) in the cecum (*p* < 0.05). Moreover, *CYP7A1* mRNA expression was negatively correlated with colonic abundances of unidentified *Clostridiales* (genus) and cecum Tenericutes (phylum). The *FXR* mRNA expression was positively correlated with *Blautia* (genus) abundance in the colon, and *NTCP* mRNA expression was positively correlated with *Holdemanella* (genus) abundance (*p* < 0.05) (Figure 11).

## 4 Discussion

Berberine (BBR) is the main active component of many herb plants including *Coptis rhizoma*, *Berberis vulgaris*, and *Cortex phellode*, and has been commonly used in the treatment of intestinal inflammation and diarrhea due to its anti-inflammatory and antibacterial properties. In recent years, increasing evidence has shown the role of BBR in regulating intestinal disorders might be closely related to modulation of gut microbiota (Wang et al., 2017; Zhang Y. et al., 2020; Zhang Z. et al., 2020), probably due to its low bioavailability in the intestine for easily interaction with gut microbiota (Wang et al., 2017). ETEC K88 is associated with increased intestinal inflammation, intestinal microbiome dysregulation, and intestinal barrier dysfunction (Fu et al., 2022; Zha et al., 2023), which accounts for the main cause for post-weaning diarrhea (PWD) in piglets. Previous study found that dietary BBR supplementation increased the growth performance and inhibited the increase of incidence of diarrhea at d 15–18 induced by ETEC challenge in weaned piglets, and the beneficial effects of dietary BBR in protecting intestinal health of weaned piglets from ETEC infection involved with the regulation of ileal microbiota and its metabolites (Zhu et al., 2023). However, it remained unclear how BBR modulate the bile acids metabolism in the gut-liver axis as well as the potential changes of microbial community in the large intestine of piglets. With the development of multiomics technology, metabolome and microbiome have been widely used to reveal the dynamic metabolic changes of intestinal microbiota and its metabolites in animals. Therefore, the combination of the targeted and non-targeted metabolome and microbiome techniques were used to investigate the profiles of bile acids in the liver and gut as well as the microbial community changes in the large intestine of piglets after treatment with BBR in this study, which aimed to reveal the potential regulative effects of BBR on the bile acid metabolism through gut-liver axis in ETEC-challenged piglets.

Bile acids constitute an important class of signal molecules that as modulators of the bidirectional dialogue between gut microbiota and the host, which play an important role in regulating host physiological functions and maintaining intestinal health (Lin et al., 2023). The liver is the critical site for primary bile acid synthesis and secretion. Briefly, bile acids are synthesized by cholesterol in hepatocytes through two different pathways: a classical pathway mediated by CYP7A1 to synthesize primary bile acids (e.g., HCA, CA, and CDCA), and an alternative pathway activated by CYP27A1 to participate in CDCA synthesis (Ridlon and Gaskins et al., 2024). Notably, the bile acid pool was significantly altered in piglets with diarrhea, which would contribute to the development of intestinal inflammation (Xu et al., 2021; Xia et al., 2022). Furthermore, increasing evidence showed that the beneficial effect of BBR on intestinal health may be related to the alterations of the composition of gut microbiota and its metabolite bile acid concentrations (Guo et al., 2016; Dehau et al., 2023a; Zhu et al., 2023). However, it remains unclear how BBR affects bile acid metabolism in the gut-liver axis of ETEC-challenged piglets. Thus, we firstly used the non-targeted metabolome techniques to seek global profiles of metabolites in liver samples of piglets, and clearly found that BBR treatment significantly altered the hepatic bile acid metabolism in ETEC-challenged piglets. Furthermore, the KEGG pathway analysis from non-targeted metabolome data showed that differential metabolites enriched in BBR group were related to the primary bile acid biosynthesis. Additional liver targeted metabolome results confirmed the enrichment and significant increase of primary conjugated bile acid changes (TCDCA and TCA) by BBR, and BBR reversed the increase of liver CA concentration induced by ETEC challenge in weaned piglets. Interestingly, previous studies have confirmed that supplementation of taurine conjugated bile acids (TCDCA and TCA) can alleviate the colitis in mice due to their anti-inflammatory properties (Wong et al., 2022). Instead, a recent study has alfo found that CA could increase inflammation in the gut and potentiate intestinal epithelial injury in colitis mice through hepatic CYP8B1-CA axis (Chen et al., 2022). Indeed, CYP8B1 is required for the classical pathway of CA synthesis of primary bile acids and mainly affects the ratio of CA to CDCA (Jiao et al., 2022). Therefore, we further determined the genes involved in bile acid metabolism in the liver, and showed that the *CYP8B1* mRNA expression in the liver was downregulated by BBR, which was in line with the decrease in hepatic CA concentration, confirming the protective effect of BBR on intestinal inflammation. Besides, our results showed that BBR treatment upregulated *CYP7A1* and *CYP27A1* expression in the liver, and promotes liver primary bile acid biosynthesis. This indicated BBR might display its beneficial effect in alleviating intestinal inflammation by regulation of bile acid metabolism in piglets followed by ETEC challenge, which was in consistent with previous study that BBR exerted its therapeutic effect by promoting bile acid synthesis (Wolf et al., 2021).

A large number of studies have shown that the bile acids-FXR axis may be an effective therapeutic target for metabolic diseases (Lin, 2019; Xiao et al., 2021). Intestinal FXR activation triggers FGF19 expression, which then returns to the liver via the portal vein to bind FGFR4, thereby represses CYP7A1 expression (Ridlon and Gaskins et al., 2024). FXR expression may be negatively related to the occurrence of inflammation, and destruction of its activity will aggravate the development of intestinal inflammation (Xiao et al., 2021; Sun et al., 2023; He et al., 2024). Furthermore, the activation of intestinal FXR expression restored bile acid metabolism homeostasis and attenuated DCA-induced intestinal inflammation in mice (Xu et al., 2021). Accordingly, current study showed that ETEC challenge significantly downregulated ileal and colonic *FXR* mRNA expression, while BBR intervention reversed the change. Consistently, previous study has shown BBR could modulate the turnover of bile acids and the FXR signal pathway in high-fat-diet-induced hamster hyperlipidemia model (Gu et al., 2015). Moreover, we showed that BBR significantly downregulated the mRNA expression of proinflammatory cytokines (*IL-1β* and *IL-8*) and upregulated the mRNA expression of ant-inflammatory cytokine (*IL-10*) in the colon of piglets challenged with ETEC. This was in consistent with previous study found in weaned piglets infected with deoxynivalenol (Tang et al., 2021) or LPS-induced IPEC-J2 cells (Zhang Z et al., 2020) after BBR interventions. Therefore, dietary BBR may reduce intestinal inflammation by inhibiting the downregulation of FXR expression and upregulation of intestinal inflammatory cytokines induced by ETEC, thereby modulating bile acids metabolism in piglets.

In order to maintain the bile acid homeostasis, about 95% of the bile acids in the intestine are reabsorbed at the end of the ileum and return to the liver through gut-liver circulation, and those unabsorbed bile acid flows into large intestine and excreted along with feces (Godlewska et al., 2022). In our previous study, the non-targeted metabolome analysis of ileal contents has showed that dietary BBR supplementation enriched the microbial metabolic pathways including primary, secondary bile acid biosynthesis and bile secretion (Zhu et al., 2023). Therefore, to better understand the potential mechanisms of BBR on bile acid metabolism for regulating the ETEC-induced intestinal inflammation. We next used the targeted metabolome technique to determine the intestinal bile acid profiles (especially the secondary bile acids) in weaned piglets fed with BBR followed by ETEC challenge. The present study showed that dietary BBR treatment could eliminate the increase of HCA, DCA, 7-KDCA and NCA concentrations in the ileum of weaned piglets induced by ETEC challenge. Notably, we also found in the BBR group significantly higher concentration of GUDCA-3S, which is conjugated from beneficial bile acids of UDCA with strong anti-inflammatory properties. Secondary bile acids are more pro-inflammatory and carcinogenic than primary bile acids due to their hydrophobicity (Collins et al., 2023). DCA has been confirmed to be the main cause of intestinal barrier damage and inflammatory responses (Xu et al., 2020; He et al., 2024). Furthermore, DCA could damage mucosal physical and functional barriers and aggravated intestinal tumorigenesis (Cong et al., 2024), and significantly increased transmissible gastroenteritis virus (TGEV) replication in swine testicular cells (Zhou et al., 2024). The beneficial effect of UDCA on physiological bile acids is due to its ability to dilute secondary toxic bile acids (DCA and LCA) to increase the concentration of beneficial bile acid pool (Winston and Theriot et al., 2020). This may explain the significantly reduction in DCA concentration after BBR intervention in the present study, thereby help restore intestinal homeostasis of weaned piglets after ETEC challenge. Furthermore, we also noticed that BBR treatment upregulated *ASBT* expression in ileum and *OST-β* expression in colon. ASBT is an important bile acid transporter for mediating the input of hepatic bile acids into the intestinal epithelial cells, while OST-β is major bile acid transporters for the output of intestinal bile acids back to the liver (Dawson et al., 2010; Nallamothu et al., 2023). In current study, the upregulated bile acid transporters of ASBT and OST-β by BBR treatment suggesting that BBR might help promote the gut-liver circulation of bile acids in weaned piglets after ETEC challenge. Previous studies have also shown BBR promotes bile acid synthesis while also increasing bile acid fecal loss to maintain the baseline hepatic bile acid concentration (Gu et al., 2015). Therefore, the protective effect of BBR on the intestine of ETEC-challenged piglets could be associated with the regulation of the biosynthesis and secretion of bile acids in gut-liver axis as well as promotion of the gut-liver circulation of bile acids.

Notably, the bile acid pool in the body is metabolically coordinated between the host and gut microbiota via deconjugation, dehydroxylation, oxidation, and epimerization (Winston et al., 2020). Thus, we next used the 16S rRNA sequencing microbiome analysis to detect the microbial changes in the large intestines of ETEC-challenged piglets fed the diets with or without BBR. The results showed that dietary BBR treatment can restore ETEC-induced intestinal microbiota dysbiosis and increased β-diversity index of large intestines in weaned piglets. Specially, at the phylum level, we found that the Firmicutes abundance was increased by BBR treatment in piglets challenged with ETEC. The phylum Firmicutes has been shown to have higher BSH activity than the phylum Bacteroidetes (Jones et al., 2008). The current study found that BBR treatment increased the relative abundance of Firmicutes, suggesting that dietary BBR intervention may increase the abundances of BSH-rich bacteria in weaned piglets. Indeed, the increase of Firmicutes would help to protect intestinal health and reduce the risk of colitis infection (Natividad et al., 2015). At the genus level, dietary BBR increased the abundances of beneficial bacteria (*Lactobacillus*, *Megamonas*, and *Blautia*) and decreased the abundances of harmful bacteria (*Prevotellaceae*, unidentified *Clostridiales*, and *Tenericutes*) in weaned piglets. Our results were also in accordance with previous study demonstrating that BBR interventions reduced the abundance of unidentified *Clostridiales* (Wultańska et al., 2020). Other researchers also found that BBR could restore the abundance of *Lactobacillus*, reduce the abundance of *Clostridium* and CA concentration to maintain intestinal homeostasis in DSS-induced colitis of mice (Hua et al., 2021). Besides, *Lactobacillus* belonging to an important group of Firmicutes with rich BSH activities, have the ability to convert conjugated bile acids into unconjugated bile acids by maintaining high levels of BSH in the gastrointestinal tract, which would help improve intestinal barrier function and prevent colitis (Deshmukh et al., 2014). Moreover, the relative abundance of the *Megamonas* was found to be negatively correlated with colonic polyps and play an important role in the development of intestinal inflammation (Ren et al., 2020). Furthermore, the potentially beneficial bacteria *Blautia* plays an important role in maintaining intestinal homeostasis and preventing inflammation (Liu et al., 2021), and involved in bile acid metabolism due to its BSH activity (Song et al., 2019). A recent study revealed that *Blautia* may be a key bacterium involved in BBR metabolism by conversion of BBR into a more readily absorbed methylated metabolite (Dehau et al., 2023b). These results confirmed the efficacy of BBR in ameliorating the gut microbiota imbalance induced by ETEC and enhancing the abundance of beneficial bacteria in piglets. Further spearman analysis showed that differential bacteria was strongly associated with alterations of bile acids in gut-liver axis. Specially, the relative abundances of Firmicutes, *Lactobacillus*, and *Blautia* were positively correlated with TCDCA. Moreover, we found that the relative abundances of unidentified *Clostridiales* and *Tenericute* were positively correlated with hepatic CA, but negatively correlated with TCDCA. Similarly, the abundance of unidentified *Clostridium* has been demonstrated positively correlated with the incidence of diarrhea and inflammatory cytokine concentrations in the ileum of piglets (Zhu et al., 2023). Indeed, *Clostridium* species are the main group of bile acid 7α-dehydroxylation (Ridlon and Gaskins et al., 2024), which could convert CA and CDCA to DCA and LCA, respectively. Besides, previous study has found that the hypocholesterolemic effect of BBR may by inhibition the bacteria with 7α-dehydroxylation activity (Gu et al., 2015). The significant reduction in ileal DCA concentration after BBR intervention in present study supported this finding. These results suggest a potential mechanism by which BBR affects bile acid metabolism by mediating the colonization of beneficial bacteria associated with bile acid metabolism and then alleviates the development of inflammation.

Importantly, bile acids might function as barrier targets for regulation of integrity of gastrointestinal mucosa (Fiorucci and Distrutti, 2015). Our results have found that colonic *Occludin* and *Claudin-5* mRNA expression were upregulated by BBR treatment in ETEC-challenged piglets. Here, we also found that BBR restored the expression of *TFF3* in the colonic mucosa after ETEC challenge in piglets. TFF3 is important markers of intestinal chemical barrier function, which could help protect intestinal mucosa from the invasion of pathogenic bacteria (Taupin and Podolsky, 2003). Previous studies have found that the expression level of *TFF3* in colonic mucosa was significantly downregulated in rats with colitis (Ritchie et al., 2017), and mice lacking TFF3 gene displayed significant delayed mucosal healing of colitis (Podolsky et al., 2009). Collectively, dietary BBR supplementation modulated the bile acid metabolism in the gut-liver axis by restoring microbial community and diversity induced by ETEC, and downregulated expression of intestinal inflammatory cytokines and upregulated the expression of genes involved in intestinal barrier function in weaned piglets challenged with ETEC.

## 5 Conclusion

In summary, the present study found that dietary BBR supplementation could restore the dysbiosis of gut microbiota and abnormal bile acid profiles induced by ETEC through modulation of the gut microbiota-bile acid-FXR axis, and significantly downregulated the expression of inflammatory cytokines while upregulating the expression of tight junction proteins to maintain intestinal homeostasis. These results might provide new insights for the development of BBR as perspective strategy for regulating the intestinal health of neonates and young piglets by targeting the microbiota-bile acid-FXR axis.

## Data availability statement

The datasets presented in this study can be found in the NCBI Bioproject repository (http://www.ncbi.nlm.nih.gov/bioproject), accession number PRJNA875031.

## Ethics statement

The animal study was approved by the Animal Care and Use Committee of Foshan University (FOSU2022005). The study was conducted in accordance with the local legislation and institutional requirements.

## Author contributions

XN: Investigation, Methodology, Writing – original draft. QL: Validation, Writing – review & editing. YY: Formal analysis, Writing – review & editing. ZH: Formal analysis, Writing – review & editing. YB: Validation, Writing – review & editing. CZ: Conceptualization, Funding acquisition, Supervision, Writing – review & editing.
