# Supplementary material for: Microbiome and metabolome analyses reveal significant alterations of gut microbiota and bile acid metabolism in ETEC-challenged weaned piglets by dietary berberine supplementation
Source: Front Microbiol. 2024 Jun 25;15:1428287. doi: 10.3389/fmicb.2024.1428287 (PMC11231202; doi:10.3389/fmicb.2024.1428287)
Supplement: Supplementary file 1 [file Table_1.DOCX]

Supplementary Material

# Supplementary Tables

**Table S1.** Composition and nutrient level of the basal diet (as fed basis)

| **Ingredients** | **Percentage, %** | **Nutrient level^1^** |  |
| --- | --- | --- | --- |
| Corn | 34.00 | ME, MJ/kg | 14.82 |
| Extruded corn | 15.51 | CP, % | 19.19 |
| Extruded soybean | 8.40 | Ca, % | 0.85 |
| Fermented soybean meal | 9.00 | Total P, % | 0.68 |
| Soybean hull | 5.00 | Available P, % | 0.50 |
| Fish meal | 4.00 | Lys, % | 1.76 |
| Whey powder | 11.00 | Met + Cys, % | 0.92 |
| Spray dried plasma protein | 4.00 | Thr, % | 1.07 |
| Soybean oil | 1.35 | Trp, % | 0.28 |
| Sugar | 2.00 |  |  |
| CaHPO_4_·2H_2_O | 1.20 |  |  |
| Limestone powder | 0.76 |  |  |
| L-Lys-HCl | 0.82 |  |  |
| DL- Met | 0.25 |  |  |
| L-Thr | 0.30 |  |  |
| L-Trp | 0.06 |  |  |
| Salt | 0.45 |  |  |
| Liquid choline chloride | 0.20 |  |  |
| Vitamin and mineral premix | 1.70 |  |  |
| Total | 100.00 |  |  |

^1^Calculated values unless indicated otherwise.

^2^Vitamin and mineral premix supplied per kilogram diet: vitamin A, 2,400 IU; vitamin D_3_, 2,800 IU; vitamin E, 200 IU; vitamin K_3_, 5 mg; vitamin B_1_, 3 mg; vitamin B_2_, 10 mg; niacin, 40 mg; vitamin B_6_, 8 mg; vitamin B_12_, 40 μg; pantothenic acid, 15 mg; folic acid, 1 mg; biotin, 0.08 mg; vitamin C, 200 mg; Fe (FeSO_4_), 120 mg; Cu (CuSO_4_), 16 mg; Mn (MnSO_4_), 70 mg; Zn (ZnSO_4_), 120 mg; I (CaI_2_O_6_), 0.7 mg; Co (CoCO_4_), 0.14 mg; and Se (Na_2_SeO_3_), 0.48 mg.
